# A sensitive and scalable fluorescence anisotropy single stranded RNA targeting approach for monitoring riboswitch conformational states

**DOI:** 10.1093/nar/gkae118

**Published:** 2024-02-20

**Authors:** Maira Rivera, Omma S Ayon, Suzana Diaconescu-Grabari, Joshua Pottel, Nicolas Moitessier, Anthony Mittermaier, Maureen McKeague

**Affiliations:** Department of Chemistry, Faculty of Science, McGill University, Montreal, QC H3A 0B8, Canada; Department of Chemistry, Faculty of Science, McGill University, Montreal, QC H3A 0B8, Canada; Department of Chemistry, Faculty of Science, McGill University, Montreal, QC H3A 0B8, Canada; Molecular Forecaster Inc. 910-2075 Robert Bourassa, Montreal, QC H3A 2L1, Canada; Department of Chemistry, Faculty of Science, McGill University, Montreal, QC H3A 0B8, Canada; Molecular Forecaster Inc. 910-2075 Robert Bourassa, Montreal, QC H3A 2L1, Canada; Department of Chemistry, Faculty of Science, McGill University, Montreal, QC H3A 0B8, Canada; Department of Chemistry, Faculty of Science, McGill University, Montreal, QC H3A 0B8, Canada; Pharmacology and Therapeutics, Faculty of Medicine and Health Sciences, McGill University, Montreal, QC H3G 1Y6, Canada

## Abstract

The capacity of riboswitches to undergo conformational changes in response to binding their native ligands is closely tied to their functional roles and is an attractive target for antimicrobial drug design. Here, we established a probe-based fluorescence anisotropy assay to monitor riboswitch conformational switching with high sensitivity and throughput. Using the *Bacillus subtillis yitJ* S-Box (SAM-I), *Fusobacterium nucleatum impX* RFN element of (FMN) and class-I cyclic-di-GMP from *Vibrio cholerae* riboswitches as model systems, we developed short fluorescent DNA probes that specifically recognize either ligand-free or -bound riboswitch conformational states. We showed that increasing concentrations of native ligands cause measurable and reproducible changes in fluorescence anisotropy that correlate with riboswitch conformational changes observed by native gel analysis. Furthermore, we applied our assay to several ligand analogues and confirmed that it can discriminate between ligands that bind, triggering the native conformational change, from those that bind without causing the conformational change. This new platform opens the possibility of high-throughput screening compound libraries to identify potential new antibiotics that specifically target functional conformational changes in riboswitches.

## Graphical Abstract

**Graphical Abstract:**
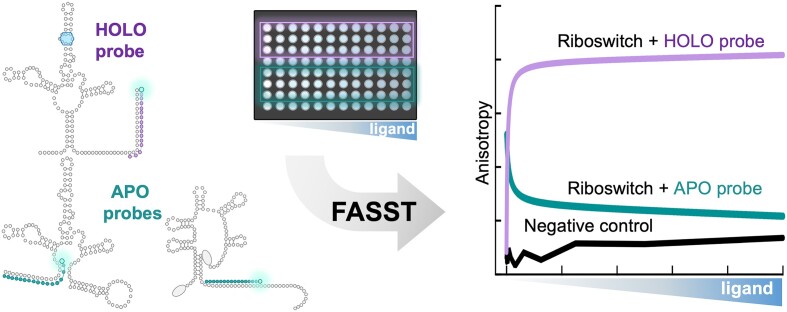

## Introduction

Riboswitches are RNA regulatory elements responsible for sensing metabolites and metal ions within cells, playing a vital role in metabolic homeostasis (1). These regulatory elements are prevalent across all domains of life and are widely distributed in bacteria, including pathogenic *Pseudomonas aeruginosa*, *Enterobacteriaceae*, *Staphylococcus aureus* and *Enterococcus faecium* (2–4). Typically located in the 5′ untranslated region of the regulated genes, riboswitches can function as either OFF or ON switches, reducing or increasing target gene expression upon sensing their ligands. Specifically, ligand binding to an aptamer domain dictates the folding of the overlapping expression platform. The expression platform then acts directly on gene expression regulating transcription, translation, or other gene expression processes through its ability to sample different conformational structures (2,5–7). Over 55 classes of riboswitches have been identified to date (2,8), and it has been proposed that many thousands of additional classes remain to be discovered among the various lineages of bacteria (2,9,10).

Due to the diversity and abundance of riboswitches in bacteria (2,11), the escalating resistance of pathogenic bacteria to antibiotics, and the lack of riboswitches found in higher eukaryotes and humans, targeting riboswitches for the development of novel antimicrobial agents has been of interest for the past two decades (12,13). Indeed, antibacterial compounds that impact riboswitch function in a manner that is unfavorable to bacteria have been described (14–16). In one example, a cell-based screen conducted at Merck & Co., identified Ribocil-C, a compound that interacts with FMN riboswitches and inhibits *Escherichia coli* growth (17). More recently, adding a second inhibitor or modifying the ribocil scaffold led to inhibition of methicillin-resistant *Staphylococcus aureus* (MRSA), *Enterococcus* faecalis, and *Klebsiella pneumoniae* (18,19). While there are currently no approved riboswitch-targeting antibiotics (3,20–22), continued efforts and interest are driving new methodologies to explore riboswitch structural regulation and screen novel ligands for antimicrobial activity.

High-throughput methods to search large libraries of candidates for potent and specific activity are necessary for drug development. For riboswitch inhibitors, different approaches have focused on ligand binding by NMR and isothermal titration calorimetry (ITC) (23), small molecule microarrays (24) or mass spectrometry (25), enabling screening of large libraries of over 50 000 compounds. However, most existing *in vitro* methods cannot discern whether binding of a given compound results in a riboswitch conformational change. Indeed, it has been shown that binding does not always result in functional switching even for molecules that differ only slightly from the natural ligand (26,27). One exception, developed by Lafontaine *et al.*, utilized molecular beacons to measure riboswitch-modulated transcriptional termination in a potentially high-throughput manner (28). However, this approach works only for the subset of riboswitches that control gene expression at the level of transcription and not for riboswitches that regulate translation (6) or mRNA stability (29). Alternatively, phenotypic screening and intracellular reporter assays can be used to assess ligand binding (28,30–33). However, the use of live cells to screen potential antimicrobials is complicated; it is difficult to determine if the effects of the inhibitors identified are due to direct interactions with the target riboswitch or through other biological mechanisms. Furthermore, some potent inhibitors may have poor uptake or retention (19,34,35) and/or a weak metabolic effect (36) preventing these from being identified in live cell assays, which omits important structure-activity information from these screens.

Given the importance of three dimensional folding to riboswitch function (37), there have been extensive efforts to characterize structural features and folding dynamics (38). For example, NMR (39), single-molecule Förster resonance energy transfer (FRET) (26,40–42), affinity labeling probes (43), SHAPE-MaP (selective 2′-hydroxyl acylation analyzed by primer extension and mutational profiling (44), and cryo-EM (45) have revealed the pathways and the kinetics of riboswitch folding in the presence and absence of native ligands (46,47). However, these methods are laborious and not conducive to the type of high-throughput screening that is necessary to identify novel ligands. Thus, new methods for the detection of ligand-mediated conformational changes in riboswitches that are both rapid and general are needed for the development of riboswitch targeting drugs.

We have developed a broadly applicable approach that leverages fluorescence anisotropy to sensitively monitor riboswitch conformational states and switching, which we term a fluorescence anisotropy single strand targeting (FASST) assay. We designed fluorescently labeled, single-stranded DNA (ssDNA) probes that can hybridize to either one of the two major conformations of the *Bacillus subtillis yitJ* S-Box (SAM-I) riboswitch. We show that gradually increasing the concentration of the natural ligand *S*-adenosyl methionine (SAM), produces clear, corresponding changes in fluorescence anisotropy. Importantly, we found that probe binding to the riboswitch is strong enough to be clearly observed, but weak enough so that perturbation of the underlying conformational equilibrium is negligible. Furthermore, ligands that bind to the aptamer but do not elicit a conformational change, show no difference in anisotropy confirming our method's specificity. To further validate our method, we applied it to the *Fusobacterium nucleatum impX* RFN element of (FMN) riboswitch, and the *Vibrio cholerae* class-I cyclic-di-GMP riboswitch, developing sensitive probes that bind specifically to the apo state of the switch and thus serves as a reporter for conformational switching in the presence of its natural ligand and established analogues. Together, our results confirm that the FASST method represents a highly specific, sensitive, and generalizable platform to detect structural rearrangements in riboswitches via easy-to-use DNA probes and fluorescence anisotropy. This new system opens the possibility for high-throughput screening to identify new potential antibiotics that specifically target the conformational change of riboswitches.

## Materials and methods

### Reagents

The following reagents were used for RNA production: Phusion HF Polymerase (#M0530S; New England Biolabs; Ipswich, MA, USA); Monarch DNA Cleanup and gel extraction kit (#T1030L; New England Biolabs; Ipswich, MA, USA); MEGAshortscript T7 Transcription Kit (#AM1354; Thermo Fisher; Waltham, MA, USA); RNA Clean & Concentrator kit (#R1018; Zymo Research; Irvine, CA, USA); and 0.025 μm MCE membrane filter disks (#VSWP02500; MF-Millipore; Burlington, MA, USA). For the surface plasmon resonance, a Biacore X100 (Cytiva Lifesciences; Marlborough, MA, USA) was used with CM5 chips (#BR100012; Cytiva Lifesciences; Marlborough, MA, USA) and HBS-N buffer 10× (#BR100828; Cytiva Lifesciences; Marlborough, MA, USA). Small molecules in the project include SAM (#A2408; MilliporeSigma; Burlington, MA, USA), SAH (#A9384; MilliporeSigma; Burlington, MA, USA), sinefungin (#S8559; MilliporeSigma; Burlington, MA, USA), FMN (#F6750; MilliporeSigma; Burlington, MA, USA), Roseoflavin (#SML1583; MilliporeSigma; Burlington, MA, USA) and Riboflavin (#47861; MilliporeSigma; Burlington, MA, USA). A BioTek Cytation 5 plate reader (#CYT5MF; BioTek Instruments; Winooski, VT, USA) with a Green Fluorescence Polarization Optical Filter Cube (#8040561; Agilent; Santa Clara, CA, USA) was used for anisotropy experiments. A Low Range ssRNA Ladder (#N0364S, New England Biolabs; Ipswich, MA, USA) and SYBR-Gold stain (#S11494; Thermo Fisher; Waltham, MA, USA) were used for the gel experiments. Finally, isothermal calorimetry was performed with a MicroCal PEAQ-ITC Automated instrument (MicroCal LLC; Malvern Panalytical, Northampton, MA, USA).

### RNA preparation

The *yitJ* SAM-I and FMN riboswitches and scrambled control RNA were produced by *in vitro* transcription from DNA templates. DNA templates were designed to contain a T7 RNA polymerase promoter sequence upstream of the riboswitch template with a 24-mer poly(A) tail at the 3′ end (Supplementary Table S1). To produce the long dsDNA templates, each sequence was ordered as four separate oligonucleotides two forward (A and B) and two reverse (C and D), from Integrated DNA Technologies (IDT) with Ultramer polyacrylamide gel electrophoresis (PAGE) purification. The oligonucleotides were amplified by PCR (1 μM each) using Phusion HF Polymerase (New England Biolabs). The resulting PCR product was verified on a 1.5% agarose gel and purified using the Monarch DNA Cleanup and gel extraction kit (New England Biolabs) following recommendations of the manufacturer.

*In vitro* transcription was performed using the MEGAshortscript T7 Transcription Kit (Thermo Fisher) with the template DNA at a final concentration of 50 μg/μl; reaction size varied between 20 to 80 μl and typically 40 μl were required for one SPR analysis run with its respective technical replicates run; 20–40 μl were required for the fluorescence anisotropy assay and 80 μl were required for ITC experiments. The mix was incubated for 16 h at 37°C. RNA was then purified by using the RNA Clean & Concentrator kit (Zymo Research). For SPR and fluorescence anisotropy assays, the RNA was quantified and stored at -20°C. For ITC measurements, the resulting RNA was then lyophilized by heating at 35°C for 40 min with 5.1 mTorr of vacuum pressure and the whole run took 1 h. Finally, the RNA was dialyzed using 0.025 μm MCE membrane filter disks (MF-Millipore) for 1 h with milli-Q water (18.2 MΩ·cm). The resulting RNA was quantified and stored at -20°C until use. Note that RNA was never used past 3 weeks of storage. The purity of the RNA was confirmed via denaturing PAGE (Supplementary Fig. S1).

### Surface plasmon resonance (SPR)

Experiments were performed as previously described (48). Briefly, SPR assays were carried out on a Biacore X100 (Cytiva Lifesciences) using a CM5 chip functionalized with a 24-poly(T). In all experiments, HBS-N running buffer (Cytiva Lifesciences; 10 mM HEPES pH 7.4; 150 mM NaCl) was supplemented with the appropriate MgCl_2_ concentration (Life Technologies). The purified RNA was prepared at 1 μM, then heated at 65°C for 10 min and finally allowed to refold at room temperature for at least ten minutes. Riboswitches were captured onto the sample flow cell (flow cell 2), while control scrambled RNA was captured on the control flow cell (flow cell 1). At least five concentrations of small molecule were injected over both flow cells at a flow rate of 30 μl/min; association and dissociation phase lengths used for each target were chosen based on time needed to reach equilibrium. RNA and small molecules were removed from the sensor surface by injecting 25 mM NaOH over both flow cells. Data analysis was performed using Biacore X100 Evaluation Software, version 2.0 (Cytiva Lifesciences). Specifically, a double-referencing method was performed to process all datasets that included first data from the sample flow cell (FC2) were referenced by subtracting data from the reference flow cell (FC1) to correct for bulk refractive index changes, nonspecific binding, injection noise, matrix effects, and baseline drift. Next, reference-subtracted data (FC2 – FC1) were double-referenced with a blank injection of running buffer to account for any systematic drift over the course of the injection. Final data were fit to a 1:1 binding model for kinetic analysis and steady-state affinity model for thermodynamic analysis where reported values are the mean and standard deviation of at least three independent experiments.

### Fluorescence anisotropy

ssDNA probes were designed to bind either apo (APO^FAM^) or holo (HOLO^FAM^) states of the riboswitches. To predict the ${T_m}$ of the probe binding to RNA, the OligoAnalyzer web tool from IDT (https://www.idtdna.com/calc/analyzer) was used, applying the following parameters for SAM-I: 150 mM NaCl, 2 mM MgCl_2_ with 50 nM probe; and for FMN: 150 mM NaCl, 5 mM MgCl_2_, with 5 nM probe. All probes were purchased from IDT with fluorescein at the 5′ end. For the SAM-I riboswitch, a modified version of the RNA sequence with an impaired expression platform was employed. In the case of the FMN riboswitch, the wild-type sequence was used, and the probe was designed to bind to the expression platform in the apo state (Supplementary Table S2).

RNA was prepared in FP buffer (10 mM HEPES pH 7.4; 150 mM NaCl; 0.05% Triton X-100) supplemented with varying concentrations of MgCl_2_ but typically 2 mM for SAM-I riboswitch and 5 mM for FMN riboswitch if not otherwise listed. Different concentrations of RNA (from 0.65 nM to 1 μM) in the absence or presence of the small molecules (200 μM SAM or 50 μM FMN) were prepared first to perform a calibration curve to confirm high affinity binding of the probe and determine the best concentration for maximum signal. For subsequence experiments with the SAM-I riboswitch, 50 nM of APO^FAM-SAM^ or HOLO^FAM-SAM^ were mixed with 75 nM and 350 nM RNA, respectively. In the case of the FMN riboswitch, 5 nM of APO^FAM-FMN^ with 350 nM RNA was used. All RNA samples were heated at 65°C for 10 min and cooled at room temperature for 10 min. Next, the probes combined with the RNA and any small molecule were loaded onto a 96-well conical black plate (Cat. No. 249945, Thermo Fisher). Equal volumes of small molecules were added in serial dilutions, ensuring that the plate was protected from light. After 30 min at 37°C, fluorescence was measured in a BioTek Cytation 5 plate reader with a green, fluorescent polarization filter (optical filter cube #8040561, Agilent). Parallel and perpendicular fluorescence were recorded every 5 min, using an excitation wavelength of 485/20 nm and an emission wavelength of 528/20 nm. Before each reading, a double orbital shake was performed for 30 s at 237 cpm (slow speed). For the SAM-I riboswitch, automatic gain at normal read speed was used. Since FMN emission/absorption is close to fluorescein, a standard dynamic range was used to fix the gain to 50 to perform the readings. Given the requirement for high concentrations of certain small molecules that may have limited solubility we also tested the impact of DMSO on our assay. As a proof-of-concept, we measured the impact of %DMSO with APO^FAM-FMN^ in the presence and absence of FMN. Fortunately, DMSO does not interfere with the fluorescence anisotropy of the assay (Supplementary Fig. S2).

Anisotropy was determined as in Equation (1), where ${I_\parallel }$ and ${I_ \bot }$ are the emitted fluorescence from samples in the parallel and perpendicular axes, respectively. ${I_\parallel }$ was determined as in Equation (2), where$\;I_\parallel ^{sample}$ is the emitted fluorescence intensity of the FAM-ssDNA probe in the parallel axis; and the $I_\parallel ^{buffer}$ corresponds to the emitted fluorescence intensity of the FP buffer in the parallel axis. This buffer correction was performed for the perpendicular fluorescence intensity to get ${I_ \bot }$. By using this approach and with increasing concentrations of different small molecules, the switching constants (50% switching), measured from this method ($S_{50}^{FASST}$) were determined by fitting a one site binding model with GraphPad Prism (version 9.0.1) as in Equation (3), where $r$ is the anisotropy value, ${r_{max}}$ is the maximum anisotropy signal, $[ L ]$ is the concentration of small molecule and $NS$ is the slope from the fit.


(1) \begin{eqnarray*}r = \left( {\frac{{{I_\parallel } - {I_ \bot }}}{{{I_\parallel } + {2_ \bot }}}} \right)\end{eqnarray*}

(2) \begin{eqnarray*}{I_\parallel } = I_\parallel ^{sample} - I_\parallel ^{buffer}\end{eqnarray*}

(3) \begin{eqnarray*}r = \frac{{{r_{max}}\left[ L \right]}}{{S_{50}^{FASST} + \;\left[ L \right]}} + NS\left[ L \right] + Background\end{eqnarray*}

### Native PAGE

RNA samples were prepared at a final concentration of 0.5 μM and mixed with different concentrations of small molecules (SAM or FMN and their analogues) followed by incubation overnight. Native gels were prepared for a Bio-Rad mini-PROTEAN system with a 1 mm width. 8% polyacrylamide:bisacrylamide (19:1) using THEM buffer (49) (34 mM Tris base, 66 mM HEPES, 0.1 mM EDTA, and the respective MgCl_2_ concentration; pH 7.5) was prepared. A 1:10 dilution of the Low Range ssRNA Ladder (N0364S, New England Biolabs) was prepared with RNA loading dye, incubated at 75°C for 5 min and immediately put on ice. Finally, 7 μl of the ladder and 10–15 μl of the prepared samples were loaded on the gel. Gels were run at 90 V at 4°C in 1× THEM buffer for 2 h 15 min, then stained using SYBR-Gold (Thermo Fisher) and imaged using ChemiDoc MP Imaging System (BioRad). For the detection of ssDNA-FAM, 10–15 μl of the mixture was loaded and run on a 10% native PAGE to detect probe binding and run under the same temperature and voltage conditions listed above.

### Isothermal titration calorimetry

Titrations of SAM and its analogues into a 10 μM RNA solution were carried out at 25°C using a MicroCal PEAQ-ITC Automated instrument (MicroCal LLC, Northampton, MA). Both samples were prepared by using a 5× buffer at a final concentration of 10 mM HEPES pH 7.4, 150 mM NaCl and 2 mM MgCl_2_. Injections were made at a rate of 0.5 μl/s and at intervals of 180 s. The first injection peak was discarded from the isotherm, as were injection peaks without a stable baseline. The baseline was generated automatically by the MicroCal PEAQ-ITC Analysis Software version 1.22 and corrected manually. Isotherms were fitted using the One Set of Sites model in MicroCal PEAQ-ITC Analysis Software version 1.22.

### Analytical description of ligand and probe binding to the riboswitches

Given the total amounts of riboswitch (${[ R ]_T}$), probe (${[ P ]_T}$), and ligand (${[ L ]_T}$), the dissociation equilibrium constants for probe binding to the apo state ($K_p^{apo}$) and holo state ($K_p^{holo}$), and an effective ligand binding constant (${K_L}$), we obtained the equilibrium concentrations of free probe ($[ P ]$), free ligand ($[ L ]$), and free riboswitch in the apo state ($[ A ]$) using non-linear least-squares regression, as described below. The effective binding constant assumes that the apo form does not bind ligand and the holo form is only formed when ligand is bound:


(4) \begin{eqnarray*}{K_L} = \frac{{\left[ A \right]\left[ L \right]}}{{\left[ {HL} \right]}},\end{eqnarray*}

and is equal to the concentration of ligand necessary to reach 50% switching (${S_{50}}$) when ${[ R ]_T}$ <<${K_L}$. The concentrations of all liganded states were calculated as follows, for the probe-bound apo state,


(5) \begin{eqnarray*}\left[ {AP} \right] = \left[ A \right]\frac{{\left[ P \right]}}{{K_P^{apo}}},\end{eqnarray*}

the ligand bound holo state,


(6) \begin{eqnarray*}\left[ {HL} \right] = \left[ A \right]\frac{{\left[ L \right]}}{{{K_L}}},\end{eqnarray*}

and holo state bound to both probe and ligand,


(7) \begin{eqnarray*}\left[ {HLP} \right] = \left[ {HL} \right]\frac{{\left[ P \right]}}{{K_P^{holo}}}.\end{eqnarray*}

The total amounts of riboswitch, ligand, and probe were calculated according to


(8) \begin{eqnarray*}\left[ R \right]_T^{sim} = \left[ A \right] + \left[ {AP} \right] + \left[ {HL} \right] + \;\left[ {HLP} \right],\end{eqnarray*}

(9) \begin{eqnarray*}\left[ L \right]_T^{sim} = \left[ L \right] + \left[ {HL} \right] + \left[ {HLP} \right]\end{eqnarray*}

(10) \begin{eqnarray*}\left[ P \right]_T^{sim} = \left[ P \right] + \left[ {AP} \right] + \left[ {HLP} \right].\end{eqnarray*}

The values of $[ A ]$, $[ L ]$, and $[ P ]$ were then varied to minimize the target function


(11) \begin{eqnarray*}F = {\left( {{{\left[ R \right]}_T} - \left[ R \right]_T^{sim}} \right)^2} + {\left( {{{\left[ L \right]}_T} - \left[ L \right]_T^{sim}} \right)^2} + {\left( {{{\left[ P \right]}_T} - \left[ P \right]_T^{sim}} \right)^2} \nonumber\\ \end{eqnarray*}

using the *fminsearch* function in MATLAB software (MathWorks).

### Statistical analyses

Statistical analyses were conducted using GraphPad Prism 9 software (GraphPad Software, San Diego, CA, USA). A one-way analysis of variance (ANOVA) was performed to assess the statistical significance of differences among multiple groups. To further investigate the differences between individual groups, post hoc multiple comparisons were performed using Dunnett's hypothesis testing, with the control group as the reference.

## Results

### Binding of SAM-I and FMN riboswitches to their natural ligands

We initially focused our attention on two well-characterized riboswitches: the *Bacillus subtillis yitJ* S-Box (SAM-I) riboswitch, which folds into two sets of helical stacks spatially arranged by tertiary interactions including a K-turn and a pseudoknot at a four-way junction (50–54) and the *Fusobacterium nucleatum impX* RFN element of (FMN) riboswitch, which consists of a six-stem junction and adopts a unique butterfly-like scaffold held together by opposing folded peripheral domains (55). Both riboswitches operate via negative feedback mechanisms, regulating the transcription of genes associated with SAM and FMN metabolism, respectively (56–61). These riboswitches are present in pathogenic bacteria such as *B. anthracis* and *L. monocytogenes* (62), making them promising targets for antimicrobial development.

**Figure 1.**
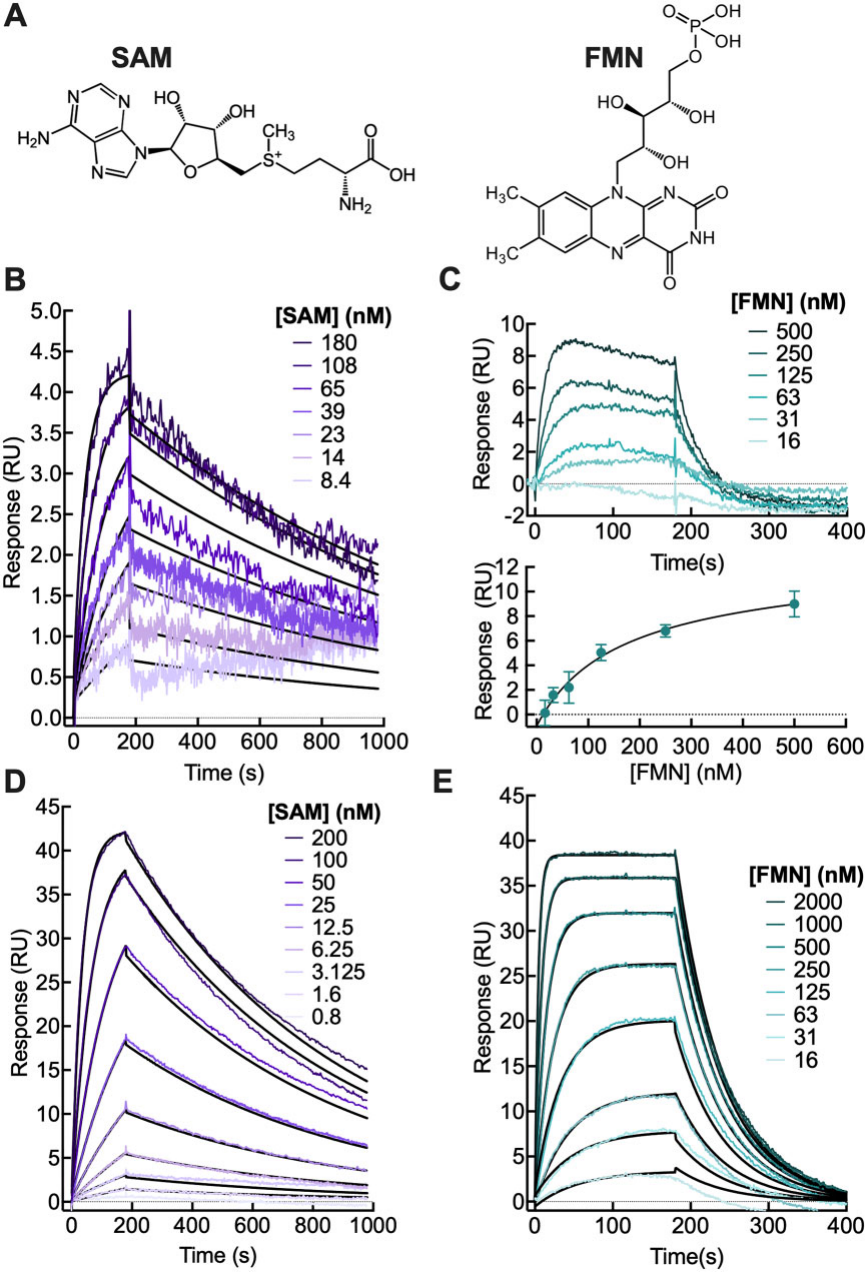
SAM-I and FMN riboswitch binding to their natural ligands measured by SPR. (**A**) Molecular structures of the native ligands *S*-adenosylmethionine (SAM) and flavin mononucleotide (FMN). (**B–E**) Sensorgrams measured using an SPR assay with association and dissociation kinetics of SAM and FMN to their respective riboswitches and the aptamer domains: (**B**) SAM-I^1-156^ (**C**) FMN^1-212^ (**D**), SAM-I^AD^ and (**E**) FMN^AD^. Note that kinetic fits are shown in black; a one binding site equilibrium fit was used for the FMN^1–212^.

We first characterized the interactions of these riboswitches with their natural ligands, using our previously developed surface plasmon resonance (SPR)-based platform (Figure 1A) (48). In all cases, the longer full-length riboswitch constructs showed similar behavior to the truncated aptamer domains, though with reduced relative response signals due to their larger masses compared to that of the ligand. Specifically, the aptamer domains of both riboswitches resulted in sensorgram responses as high as 40 relative response units (RU) compared to a maximum of 4 and 10 RU for the full SAM-I and FMN riboswitches respectively (Figure 1). Binding was in the slow kinetic regime; the ligand capture and release curves were fitted to obtain association and dissociation rate constants. The ratio of these two values gave reproducible apparent equilibrium dissociation constants, $K_D^{ligand}$, for all riboswitches tested. The full length (residues 1–156) and aptamer domain (residues 6–126) of the SAM-I riboswitch bound SAM with similar, $K_D^{ligand}$ values of 10.1 ± 0.4 and 4 ± 2 nM, respectively (Figure 1B and D). These values are consistent with the nM affinities reported in the literature using filtration experiments and in-line probing (50,56,63) and in solution measuring using isothermal titration calorimetry (ITC, Supplementary Fig. S3). Similar observations were made for the FMN riboswitch (Figure 1C and E), resulting in calculated $K_D^{ligand}$ values of 95 ± 50 nM for the full-length riboswitch (residues 1–212) and 160 ± 12 nM for the aptamer domain (residues 21–127). These are comparable with previously-reported values that range from 7.5 to 74 nM based on fluorescence (55), in-line probing (64) and SPR assays (65).

### Designing DNA probes to detect riboswitch conformational changes.

**Figure 2.**
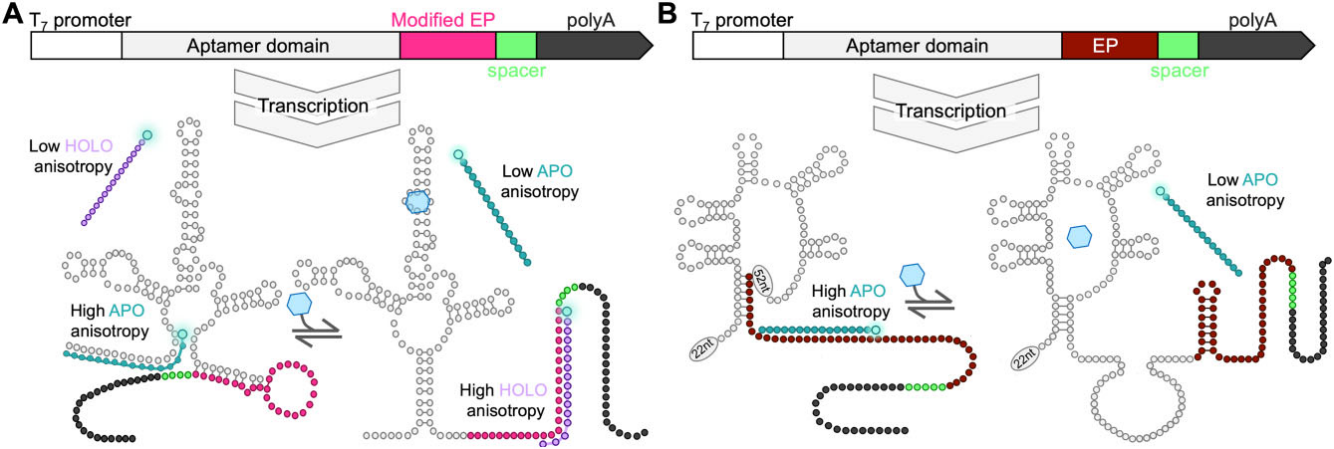
Design of SAM-I and FMN riboswitches and fluorescence anisotropy approach for sensing riboswitch conformational change. (**A**) The SAM-I riboswitch was designed with a partially truncated expression platform (magenta) to enable the hybridization of a DNA probe in the SAM-bound state (purple probe; HOLO). (**B**) The FMN riboswitch was designed with its complete expression platform (dark red). Both constructs include a spacer and a poly A tail, which were utilized for the SPR assays. Fluorescently labeled DNA probes, designed to bind to the apo state (teal probes; APO; A and B), were developed for both riboswitches. A pair of probes will show opposite behavior upon increasing concentrations of the riboswitch target. HOLO^FAM^ results in low anisotropy measurements due to a depolarized emission of the free probe. Upon higher concentrations of ligand, fluorescence of the probe becomes more polarized as it binds to the larger riboswitch construct.

In general, transitions from apo to holo riboswitch forms are accompanied by the unfolding of certain RNA structures and the formation of others. As a result, there are regions of RNA that are single-stranded in the apo state and double-stranded in the holo state, or vice-versa. We designed short DNA probes that specifically hybridize to these regions when exposed in either the apo- or holo-state and are displaced when these same regions form RNA duplexes in the opposite state. For example, the 5′ end of the SAM-I riboswitch is single-stranded in the apo state and forms an RNA stem in the holo state. A DNA probe complementary to this region can in principle bind to the apo state but not the holo state. Conversely, the 3′ end of our SAM-I riboswitch construct forms part of an RNA hairpin in the apo state and is single-stranded in the holo-state (Figure 2A). Note that the naturally occurring SAM-I riboswitch is 13 residues longer which leads to formation of a terminator hairpin at this location in the holo state. We designed a slightly truncated version to ensure that it remains single-stranded; a DNA probe that is complementary to this 3′ region can, in principle, hybridize with the holo state, but not the apo state (Figure 2A). The same design approach was applied to the FMN riboswitch (Figure 2B). Thus ligand-induced conformational changes should be accompanied by either DNA probe hybridization (HOLO probe) or displacement (APO probes). To monitor these changes in a simplistic manner, the probes included a fluorescein modification at the 5′ end and we measured steady-state fluorescence anisotropy (Supplementary Fig. S4). When a probe in the unbound state is excited with polarized light, rapid tumbling in solution leads to a low degree of polarization of the emitted light, resulting in close-to-zero fluorescence anisotropy values (Figure 2). Conversely, when the probe is bound to the much larger riboswitch, the rate of tumbling is slower, and the emitted light retains more of the initial polarization. Thus, probe binding to the riboswitch is detected as an increase in fluorescence anisotropy, while conformational changes that cause the probe to dissociate lead to decreases in fluorescence polarization, a process we refer to as fluorescence anisotropy single stranded targeting (FASST). Based on structural data available for the SAM-I riboswitch (50–54) we designed FASST DNA probes to specifically recognize both apo and holo states (APO^FAM-SAM^ and HOLO^FAM-SAM^). Less is known regarding the conformational changes occurring in the FMN riboswitch, so only one probe was designed to hybridize with the expression platform in the apo state (APO^FAM-FMN^).

To verify the selectivity and optimize the efficacy of the three probes, we first determined their binding affinities with the target riboswitches in the presence and absence of native ligand. Probe concentrations were held constant while riboswitch RNA was titrated in, monitoring fluorescence anisotropy (Supplementary Fig. S5). The extracted apparent binding constant ($K_D^{probe})\;$for SAM-I with the APO^FAM-SAM^ probe in the absence of SAM was 73 ± 14 nM while for the HOLO^FAM-SAM^ probe with saturating SAM, $K_D^{probe}$>350 nM. The APO^FAM-FMN^ probe bound the FMN riboswitch in the absence of FMN with an apparent binding constant ($K_D^{probe})$ of 340 ± 46 nM. No detectable binding was observed for the HOLO^FAM-SAM^ probe in the absence of SAM nor the APO^FAM-FMN^ probe in the presence of FMN, as hoped. The APO^FAM-SAM^ did also bind the riboswitch in the presence of SAM, albeit with an affinity reduced by about 2-fold. To better understand this unintended interaction between the APO^FAM-SAM^ probe and holo state of the riboswitch, we examined the 5′ end of the riboswitch. This region is single-stranded in the apo form and complementary to the APO^FAM-SAM^ probe. In the holo form, most of the 5′ end forms a long stem, which we had anticipated would prevent binding of the probe. However, there remain five single-stranded residues at the 5′ end that could potentially still allow the probe to bind and possibly invade the stem structure to some degree. Previous work indicates that the long stem of the aptamer domain does not need to be completely formed in the binding competent state (40). We tested APO^FAM-SAM^ binding to a version of the riboswitch lacking these five terminal residues and found that it does not bind, either in the presence or absence of SAM, confirming that the APO^FAM-SAM^ probe binds the same regions of the riboswitch in both apo and holo forms (Supplementary Fig. S6). Our design had intended that the formation of the stem in the holo form would completely block APO^FAM-SAM^ binding, but instead merely weakened probe binding. Fortunately, this difference in affinity for the apo and holo forms was still sufficient to detect conformational switching using this probe. We then tested different amounts of probe and riboswitch, over timepoints ranging from 5 minutes to 24 hours, and at MgCl_2_ concentrations ranging from 2 and 5 mM, seeking to optimize the difference in probe fluorescence anisotropy between the ligand bound and free states. We found that robust signals could be obtained with probe concentrations as low as 5 nM. Empirically, for the SAM-I probes, the strongest signal to noise was obtained with probe concentrations of 50 nM, while for the APO^FAM-FMN^, 5 nM was determined to be sufficient. The optimal concentration of riboswitch was found to be approximately equal to the probe $K_D^{probe}$. Under these conditions, the riboswitch binds at most 50% of the corresponding probe and small changes in binding constants produce large changes in the amount of probe that is bound. The optimal conditions determined here (Table 1) were applied for the subsequent structural switching assays.

**Table 1.** Conditions for fluorescence anisotropy measurements

Probe	Riboswitch	Incubation time	$K_D^{probe}$	[RNA]	[Probe]	MgCl_2_
HOLO^FAM-SAM^	SAM-I^1–156^	3 h	>350 nM	350 nM	50 nM	2 mM
APO^FAM-SAM^	SAM-I^1–156^	45 min	73 ± 14 nM	75 nM	50 nM	2 mM
APO^FAM-FMN^	FMN^1–212^	3 h	340 ± 46 nM	350 nM	5 nM	5 mM
APO^FAM-c-di-GMP^	c-di-GMP^1–209^	3 h	70 ± 12 nM	70 nM	50 nM	5 mM

Based on our results, we propose a set of guidelines for designing new ssDNA probes for riboswitches of interest and for applying them to the FASST assay.

- Probes should target single stranded RNA regions that are exclusively present in either the apo or in the holo folded state. Despite that riboswitch structural data focus on the study of their aptamer domain, potential single stranded regions can be identified using a combination of structural information such as X-ray crystallographic structures or experimental secondary structural data from SHAPE, along with secondary structure prediction programs. In some cases, web servers like Rfam (66) can provide a valuable resource by assisting in the identification of riboswitch families with similar sequence identity. For instance, potential single-stranded and switching regions can be predicted through homology by comparing riboswitch structures to other members with well-described expression platforms. In general, it is crucial to gather as many data as possible to refine the potential single stranded regions for probe design targeting.
- Riboswitches can be slightly modified to generate suitable single stranded binding sites for fluorescent probes. We find that the expression platform is a productive region in which to make these modifications. In transcriptional riboswitches, the expression platform forms a double stranded hairpin terminator that blocks RNA polymerase transcription. Similarly, in translational riboswitches, formation of a hairpin blocks with the Shine-Dalgarno (SD) sequence, preventing translation. Removal of half of the hairpin in the expression domains leaves positions for probes to bind instead. In this way, the probe binding mimics the full natural expression domain (see Supplementary Fig. S7). The holo SAM and apo FMN probes were designed using this strategy.
- When the single-stranded RNA regions are identified, complementary ssDNA probes should be designed such that the melting temperatures range between about 35°C to 40°C, avoiding intramolecular secondary structures. Our probes ranged from 11 to 16 nucleotides in length.
- With probes in hand, optimal binding conditions can be determined by performing an RNA calibration curve at more than one constant probe concentrations (here we used 5 and 50 nM), in the presence and absence of high concentrations of the riboswitch native ligand. The switching assay can then be performed using the lowest probe and RNA concentrations that give the maximum change in anisotropy upon probe binding to the RNA. Proper controls include the probe with ligand in the absence of the RNA to account for any non-specific interactions of ligand. Finally, measuring the switching over several time points (5 min to several hours) is useful given that certain probes may exhibit slower binding kinetics, or the ligand may act as a delayed switching trigger.

### Quantifying riboswitch conformational switching via fluorescence anisotropy

**Figure 3.**
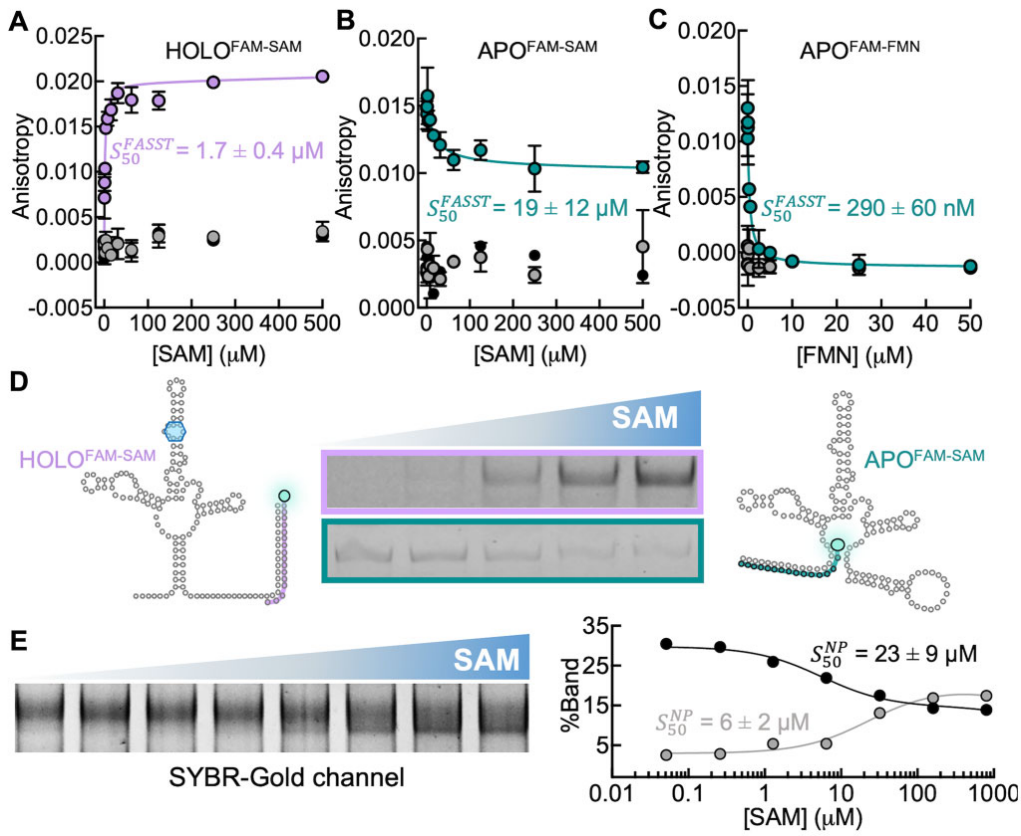
Fluorescein labeled ssDNA probes hybridize to specific conformations of the SAM-I and FMN riboswitches and sense conformational switching. (A–C) Binding of the probes measured using fluorescence anisotropy upon increasing concentrations of the riboswitch native ligand. An increase in anisotropy represents binding of the probe to the riboswitch whereas a decrease in anisotropy represents the displacement of the probe from the riboswitch. The change in anisotropy vs concentration was used to fit switching constants $S_{50}^{FASST}$. (**A**) HOLO^FAM-SAM^ with the SAM-I riboswitch, $S_{50}^{FASST}$= 1.7 ± 0.4 μM (**B**) APO^FAM-SAM^ with the SAM-I riboswitch, $S_{50}^{FASST}$= 19 ± 12 μM (**C**) APO^FAM-FMN^ with the FMN riboswitch, $S_{50}^{FASST}$= 290 ± 60 nM. (**D**) Native PAGE of the HOLO^FAM-SAM^ and APO^FAM-SAM^ probes binding and releasing from the SAM-I riboswitch upon incubation with increased concentrations of SAM. (**E**) Native PAGE of the SAM-I riboswitch with increasing concentration of SAM in the absence of probes and the resulting fit for two bands in the gel. Top band (black) $S_{50}^{NP}$= 6 ± 2 μM and bottom band (gray) $S_{50}^{NP}$ were and 23 ± 9 μM. (A–C) Error bars represent the standard deviation of duplicate experiments black dots correspond to the probes in the presence of SAM or FMN and grey dots correspond to the probe in the presence of a scrambled RNA and SAM or FMN.

We next set out to characterize ligand-induced conformational changes in the SAM-I and FMN riboswitches using the fluorescent probes described above. SAM, at concentrations between 0 and 500 μM, and FMN, at concentrations between 0 and 50 μM, were incubated with riboswitches and probes at the concentrations listed in Table 1. In parallel, each probe was also incubated with the native ligand in the absence of riboswitch, as negative controls. As expected, the HOLO^FAM-SAM^ probe initially showed a low fluorescence anisotropy value close to that of the negative control. Increasing anisotropy was observed with increasing concentrations of SAM, confirming the idea that it specifically recognizes the ligand-bound conformation of the riboswitch (Figure 3A). To obtain a switching constant ($S_{50}^{FASST}$, the concentration of ligand necessary to reach 50% switching measured via this method) of 1.7 ± 0.4 μM (Table 2), we fit a one site binding model to the fluorescence anisotropy data with Graphpad Prism (as described in Methods). In contrast, the APO^FAM-SAM^ for SAM-I riboswitch initially exhibited high anisotropy which decreased with increasing concentrations of SAM (Figure 3B), in line with the idea that the probe can no longer bind to the SAM-bound state. In the presence of the ligand, fluorescence anisotropy did not decrease to the negative control values, consistent with our observation that the APO^FAM-SAM^ probe maintains some, albeit weaker, affinity for the holo state. Previous work indicates that the long stem of the aptamer domain does not need to be completely formed in the binding competent state (40) providing a possible shorter single-stranded binding sequence for the apo probe in the holo state. The calculated switching constant was similar to that of the HOLO^FAM-SAM^ probe ($S_{50}^{FASST}$=19 ± 12 μM) confirming that the pair of conformational probes are monitoring the same riboswitch conformational transition. Furthermore, to better understand the impact of the poly(A) tail on the folding and binding capability of the SAM-I riboswitch, an additional control was performed with an RNA lacking the spacer and the poly(A) tail described in Figure 2A. As a result, we obtained $S_{50}^{FASST}$ values of 0.19 ± 0.08 μM and 8 ± 3 μM for HOLO^FAM-SAM^ and APO^FAM-SAM^, respectively (Supplementary Fig. S8). These results suggest that the poly(A) tail, intended for SPR experiments, does not affect the binding and switching measured by fluorescence anisotropy in solution. For the FMN riboswitch, a decrease in anisotropy was measured as the concentration of FMN increased, confirming that binding of the probe is blocked by the ligand-induced switching of the riboswitch. A $S_{50}^{FASST}$ value of 290 ± 60 nM was calculated for the APO^FAM-FMN^ (Figure 3C) which is similar to the value of $K_D^{ligand}$ measured by SPR.

**Table 2.** Binding affinity and switching parameters of the riboswitches

Riboswitch	Ligand	$K_D^{ligand}$ ^a^	$S_{50}^{FASST}$	$S_{50}^{NP}$
SAM-I^1-156^	SAM	10.1 ± 0.4 nM	1.7 ± 0.4 μM^b^ 19 ± 12 μM^c^	6 ± 2 μM^d^ 23 ± 9 μM^e^
	SAH	46 ± 27 μM	N/S	N/S
	Sinefungin	2 ± 3 mM	N/S	N/S
FMN^1-212^	FMN	150 ± 180 nM	290 ± 60 nM	N/A
	Riboflavin	32 ± 11 μM	0.7 ± 0.3 μM	N/A
	Roseoflavin	4.9 ± 0.2 μM	24 ± 20 μM	N/A
c-di-GMP^1-209^	c-di-GMP	N/A	54 ± 22 nM	22 ± 12 nM^f^ 3 ± 5 nM^g^

^a^Apparent ${K_D}$ measured by SPR.

^b^ ${S_{50}}$ measured with HOLO^FAM-SAM^.

^c^ ${S_{50}}$ measured with APO^FAM-SAM^.

^d^ ${S_{50}}$ measured by native page (NP) with top band on Figure 3E.

^e^ ${S_{50}}\;$ measured by NP with bottom band on Figure 3E.

^f^ ${S_{50}}\;$ measured by NP with bottom band on Supplementary Figure S9D.

^g^ ${S_{50}}$ measured by NP with top band on Supplementary Figure S9D.

All riboswitches underwent testing for binding and switching with their natural ligands or analogues. Switching tested via the FASST method was compared to that obtained via native gel electrophoresis. N/A means the experiment was not performed, and ‘N/S’ indicates the absence of switching detected under the tested experimental conditions.

To validate the fluorescence anisotropy measurements, we characterized the interactions of the fluorescent probes with the SAM-I riboswitch using native PAGE. As the concentration of SAM increased in samples containing the riboswitch and the HOLO^FAM-SAM^ probe, a corresponding increasing in band intensity was observed at the switch band. In contrast, for samples containing the riboswitch and the APO^FAM-SAM^ probe, fluorescence at the riboswitch band decreased with increasing concentrations of SAM (Figure 3D). This is consistent with the HOLO^FAM-SAM^ and APO^FAM-SAM^ probes specifically recognizing the ligand-bound and -free forms of the SAM-I riboswitch, respectively. We analyzed the PAGE pixel densities and obtained switching constants ($S_{50}^{NP})$ with similar values to the switching constants $( {S_{50}^{FASST}} )$ derived from fluorescence anisotropy measurements: 1.03 ± 0.08 and 46 ± 44 μM for HOLO^FAM-SAM^ and APO^FAM-SAM^, respectively (Supplementary Figure S9).

Given the large (two orders of magnitude) differences between the binding constants ($K_D^{ligand}$) obtained from SPR and the switching constants $(S_{50}^{FASST})$ obtained from fluorescence measurements for the SAM-I riboswitch, we investigated ligand-induced conformational changes in the absence of probes in greater detail using native PAGE. This method has previously been shown to separate different riboswitch conformational states based on to their differing degrees of compaction which lead to different migration rates (67,68). In the absence of SAM, the main riboswitch band migrated on a polyacrylamide gel with an effective size of about 500 bases. As the concentration of the SAM ligand increased, a second band with faster migration appeared on the gel in a concentration-dependent manner. We analyzed the pixel densities of both bands, plotting both the disappearance of the top band and the appearance of the lower band, as functions of SAM concentration. From these data, we calculated switching constants ($S_{50}^{NP}$) in the low μM range (6 ± 2 μM for the top and 23 ± 9 μM for the bottom) (Figure 3E). These values are in remarkably good agreement with the values obtained from fluorescence anisotropy and validate that the fluorescent probes are sensing the conformational changes in the riboswitch. The source of the discrepancies between $S_{50}^{FASST}$ and $K_D^{ligand}$ are currently unknown and the focus of ongoing investigation in our labs and are discussed in more detail below.

### Riboswitch, ligand and probe binding equilibria

**Figure 4.**
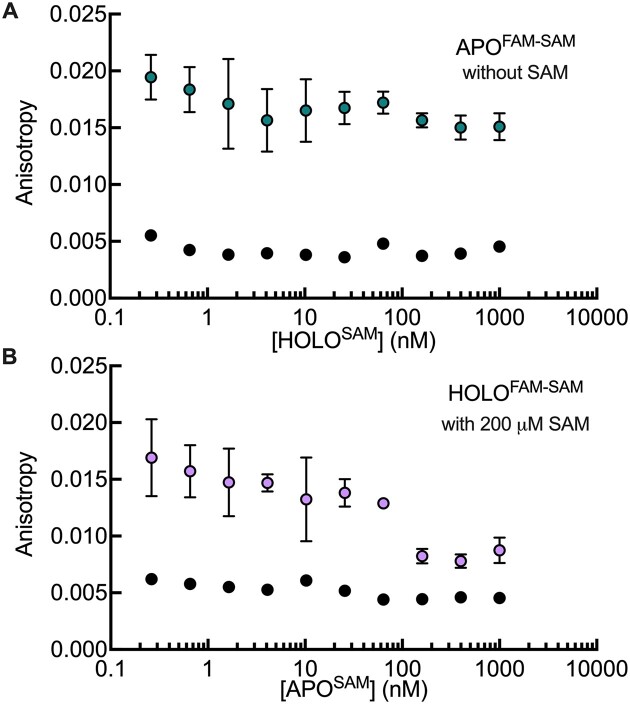
Impact of probes on SAM-I riboswitch conformational switching. (**A**) Binding of the APO^FAM-SAM^ probe in teal to 75 nM of SAM-I riboswitch RNA in the presence of increasing concentrations of the opposite unlabeled HOLO^SAM^ ssRNA measured via fluorescence anisotropy over time. (**B**) Binding of the HOLO^FAM-SAM^ probe in purple to 350 nM SAM-I riboswitch RNA, in the presence of increasing concentrations of the opposite unlabeled APO^SAM^ ssDNA. The controls (black) are the fluorescent probes in the presence of the unlabelled probes without any riboswitch RNA. An increase in anisotropy compared to the control indicates binding of the labelled probes to the SAM-I riboswitch. Error bars represent the standard deviation of duplicate experiments.

The FASST method involves complex and interconnected binding and conformational equilibria, therefore it is imperative that we explore how the binding and release of fluorescent probes relate to the ligand-induced switching behaviour of interest. One important question is how the addition of fluorescent probes may alter the switching equilibrium itself. According to Le Chatelier's principle, any probe that binds specifically to the apo (or holo) state will tend to shift the population of RNA structures towards that state. To examine this phenomenon, we employed our two opposing probes for the SAM-I riboswitch that bind preferentially to either the ligand-bound or ligand-free conformational states. The binding of each labelled probe (HOLO^FAM-SAM^ and APO^FAM-SAM)^ was measured in the presence of increasing concentrations of the unlabelled opposite probe (APO^SAM^ and HOLO^SAM^). When the SAM-I riboswitch and the APO^FAM-SAM^ probe, at the concentrations listed in Table 1, were incubated with unlabeled HOLO^SAM^ ssDNA overnight at concentrations up to 1 μM, we saw modestly decreasing fluorescence anisotropy values at higher concentrations of the unlabelled ssDNA, suggesting that its presence slightly shifts the conformational equilibrium of the riboswitch towards the holo state, as expected (Figure 4A). Conversely, when the riboswitch, HOLO^FAM-SAM^ probe, and 200 μM SAM were incubated overnight with increasing concentrations of the unlabeled APO^SAM^ ssDNA, decreasing fluorescence anisotropy values were obtained with increasing concentrations of the APO ssDNA, with the effect particularly pronounced above about 160 nM (Figure 4B). These observations were confirmed through a one-way ANOVA statistical analysis (multiple comparison test), revealing significant differences between HOLO^FAM-SAM^ fluorescence anisotropy in the absence of APO^SAM^ and concentrations of APO^SAM^ equal or greater than 160 nM (*P* ≤ 0.001). Thus, the APO^SAM^ ssDNA shifted the riboswitch conformational equilibrium towards the apo form at higher concentrations. These observations confirm that care must be taken to avoid high probe concentrations that could perturb switching equilibria. Accordingly, we performed our assays with relatively low probe concentrations and excesses of riboswitch to minimize this effect. For example, in the FMN riboswitch assay, the concentration of probe was 5 nM, while that of the riboswitch was 350 nM. Under these conditions, which correspond to RNA concentrations below the apparent binding constants of the probes to the riboswitch ($K_D^{probe})$, there are not enough probe molecules to cause any significant shifts in the populations of apo and holo states.

Another important question is how accurately the binding of the fluorescent probes reports on the apo/holo equilibrium. The fact that $S_{50}^{FASST}$ values derived from the FASST assay and native gel electrophoresis experiments are comparable provides some degree of reassurance. However, to address this question more quantitatively, we calculated estimates of the amounts of all ligand, probe, and riboswitch species present during the experiments based on the concentrations of reagents used and the measured affinities of the probes. The fact that ligands, probes, and riboswitches are present at concentrations similar to their equilibrium dissociation constants means that there is no simple equation for directly determining the extents of binding. Fortunately, this problem can be solved numerically (see Materials and methods section). Based on these calculations, when the probe is in large excess of the riboswitch, the apo/holo equilibrium is substantially perturbed, as discussed above. Nevertheless, the switching curve observed experimentally by fluorescence anisotropy exactly matches the (perturbed) conformational equilibrium (Supplementary Figures S10 and S11). Conversely, when the riboswitch is in large excess of the probe, the riboswitch conformational equilibrium is not perturbed. However, the extent of fluorescent probe binding is no longer proportional to the extent of conformational switching. As a result, the experiments tend to overestimate ${S_{50}}$ values for APO probes and underestimate ${S_{50}}$values for HOLO probes. The deviation is about a factor of two when the riboswitch concentration is equal to $K_D^{probe}$ and is larger or smaller when the riboswitch concentration is higher or lower, respectively. The underlying reason is that when the probe concentration is low, the free probe becomes substantially depleted in solution when the probe-binding riboswitch conformation is plentiful (in the absence of ligand for APO probes and in the presence of ligand for HOLO probes). As a result, binding is relatively less favoured than when the probe-binding riboswitch conformation is scarce and the amount of free probe in solution is higher. Finally, when the riboswitch and probe are roughly equal in concentration, the switching equilibrium is slightly perturbed and the probe binding is shifted slightly relative to the conformational switching, with the net effect that experimentally observed $S_{50}^{NP}$ values differ from the true, unperturbed values; when the concentrations of probe and riboswitch are about equal to the $K_D^{probe}$, the deviation is also about two-fold. What this implies is that when the riboswitch concentration is equal to or less than the $K_D^{probe}$, these experiments are relatively insensitive to the amount of probe added, up to about an equimolar ratio, and that $S_{50}^{NP}$ values are overestimated for APO probes and underestimated for HOLO probes by, at most, about a factor of 2. We simulated the titrations for the four different probes used in this study under the experimental conditions used and estimate that deviations between observed and true ${S_{50}}$ values range from about factors of 1.3 to 1.9 (Supplementary Figure S12).

Finally, it is worthwhile considering the situation where a probe binds to both apo and holo forms of a riboswitch, as we observe for the APO^FAM-SAM^ probe. Provided that the affinities for the two forms are different, such molecules can still be effective probes of conformational switching. The two main ramifications are firstly, perturbations of the switching equilibrium are reduced since the equilibrium shifts induced by binding to apo and holo forms partially cancel each other out. Secondly, the overall sensitivity of the experiment is substantially reduced, since there are smaller changes in the concentration of free probe during a ligand titration. According to our calculations (Supplementary Figure S13), the sensitivity is reduced by about 50 to 60% when the ratio of the apo and holo affinities is 10 and reduced by 85 to 90% when the ratio of the affinities is 2. Furthermore, the sensitivity of the experiment becomes more strongly dependent on the concentration of riboswitch, than for probes that bind uniquely to one state. We find that optimal sensitivity is achieved when the riboswitch concentration is roughly equal to the $K_D^{probe}$ values of the probe for the apo and holo states, or to the probe concentration itself, whichever is larger.

### Discriminating ligand binding from conformational switching

**Figure 5.**
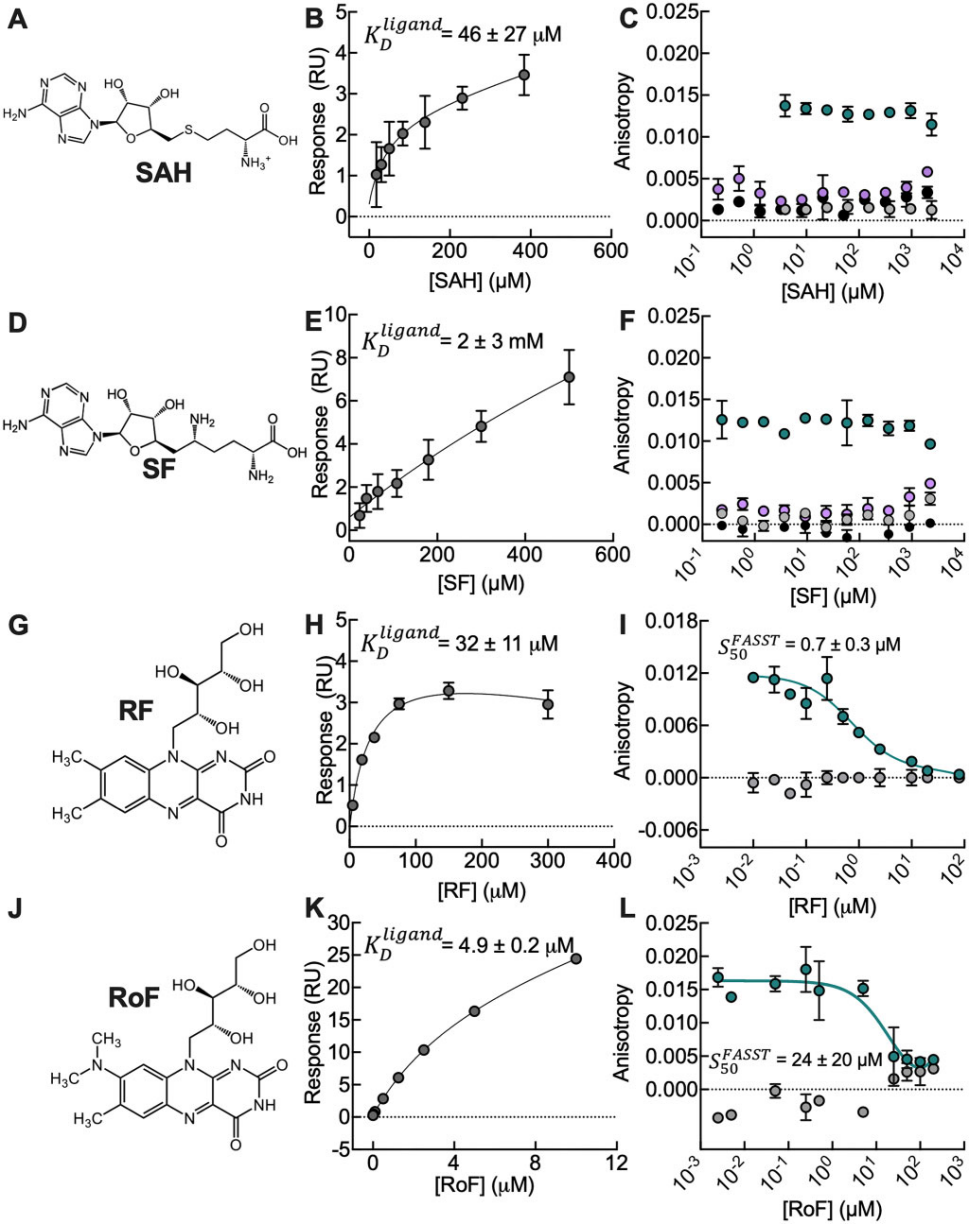
Analogue binding to riboswitches does not always result in riboswitch conformational changes. (**A, D**) SAH and sinefungin (SF) structures, (**B, E**) SPR affinity fit of SAH and SF to the SAM-I riboswitch, (**C, F)**, Fluorescence anisotropy assay for SAH and SF with the SAM-I riboswitch using the APO^FAM-SAM^ probe in green and the HOLO^FAM-SAM^ in purple compared to the probes alone (APO^FAM-SAM^ control in gray; HOLO^FAM-SAM^ control in black). (**G, J**) Riboflavin (RF) and roseoflavin (RoF) structures, (**H, K**) SPR affinity fit of RF and RoF to the FMN riboswitch, (**I, K**), Fluorescence anisotropy assay for RF and RoF to the FMN riboswitch using the APO^FAM-FMN^ probe in green compared to the corresponding probe in buffer (gray). Error bars represent the standard deviation of duplicate experiments. SAM-I riboswitch experiments were performed at 2 mM MgCl_2_, and FMN riboswitch experiment were performed at 5 mM MgCl_2_ (Table 1).

Towards our goal of leveraging the FASST assay to screen riboswitch targeting molecules that directly result in functional conformational changes, we next investigated a panel of ligand analogues that bind to the SAM-I and FMN riboswitches. For instance, S-adenosyl homocysteine (SAH) binds the SAM-I riboswitch with a 100-fold lower affinity than SAM itself (56,69), but it only triggers the structural switch required for gene regulation under certain solution conditions (56). SAH was therefore an ideal model ligand to test whether our method can discriminate ligand-induced structural switching apart from aptamer binding alone. We first confirmed binding of SAH to the SAM-I riboswitch using the same SPR assay described above. The association and dissociation kinetics of SAH binding to the riboswitch were too fast for kinetic fitting, therefore, the steady state binding was used to calculate the affinity (Figure 5B). As expected, SAH binding was weak compared to the SAM-I native ligand SAM, with a $K_D^{ligand}$ of 195 ± 22 μM for the aptamer domain (Supplementary Table S3) and 46 ± 27 μM for SAM-I^1-156^, consistent with previously published values of about 400 μM (56,69). We next employed our FASST assay to test for riboswitch conformational changes in the presence of SAH. The APO^FAM-SAM^ and HOLO^FAM-SAM^ probes produced consistently high and low fluorescence values, respectively, even at SAH concentrations 10-fold larger than $K_D^{ligand}$ and overnight pre-incubation, indicating that the SAM-I riboswitch retains the apo conformation, even when bound to SAH under our experimental conditions (Figure 5C). At high mM SAH concentrations a slight change in anisotropy was observed that could indicate switching at higher concentrations of SAH. Considering that magnesium stabilizes the aptamer domain of SAM-I riboswitch, we performed the same experiment with SAH at 5 mM MgCl_2_. Some switching at high concentrations of SAH was detected with the HOLO^FAM-SAM^ probe, consistent with previous studies (26). This weak switching at high concentrations of SAH and magnesium was corroborated via native PAGE analysis in the absence of the probe (Supplementary Figure S14). The modest changes in anisotropy with SAH at mM concentrations suggests that there are switching events occurring and therefore full switching events at even higher concentrations cannot be discarded. Together, our data suggest that our FASST method is sensitive enough to determine when the SAM-I riboswitch functional conformational change is induced by ligand binding and when it is not.

We also analyzed additional analogues that have been previously found to bind to SAM and FMN riboswitches. Sinefungin (SF) is a SAM analogue that contains ornithine in place of methionine and binds to the SAM-I riboswitch with an affinity 500-fold weaker than that of SAM. Its impact on expression is unclear (58,69). Riboflavin (RF) and roseoflavin (RoF) are two naturally occurring FMN analogues that have been reported to bind to the FMN riboswitch and control the expression of downstream genes. Specifically, riboflavin lacks a phosphate group present in the native ligand while roseoflavin both lacks the phosphate group and substitutes the methyl group at position 8 with a dimethylamino moiety (70). According to our SPR measurements, all these compounds showed weaker affinities for the corresponding riboswitches compared to the native ligands with $K_D^{ligand}$ values of 2 ± 3 mM for sinefungin, 32 ± 11 μM for riboflavin, and 4.9 ± 0.2 μM for roseoflavin (Figure 5E,H,K), according to our SPR measurements. These values are both consistent with the literature and with $K_D^{ligand}$ values measured for the aptamer domains by SPR (Supplementary Fig. S15 and Supplementary Table S3).

We employed our FASST assay to measure any possible riboswitch conformational changes in the presence of these analogues. Similarly to SAH, no structural switch of the SAM-I riboswitch was observed with sinefungin even at high concentrations and with overnight incubation (Figure 5F), supporting earlier work where sinefungin failed to promote transcription termination (58). However, both riboflavin and roseoflavin resulted in significant conformational switching following an overnight incubation, with a $S_{50}^{FASST}$= 0.7 ± 0.3 μM for riboflavin (Figure 5I) and $S_{50}^{FASST}$= 24 ± 20 μM for roseoflavin (Figure 5L). These results support the use of these FMN analogues as antimetabolites and inhibitors that target the FMN riboswitch and exert antibacterial effects (17). Notably, riboflavin exhibits a $S_{50}^{FASST}$ lower than binding affinity determined by SPR, this discrepancy could arise from a potential overestimation of the $K_D^{ligand}$ of riboflavin. Indeed, previous studies with the FMN riboswitch from *B. subtilis*, with affinities close to 3 μM derived from in-line probing experiments (16,60), are closer to our switching values.

SAM-I and FMN riboswitches are transcriptional riboswitches; therefore, we aimed to assess the generalizability of our method to a translational riboswitch. We designed a ssDNA probe capable of binding to the apo state of the class-I cyclic-di-GMP from *Vibrio cholerae* (71). In particular, the probe was designed to interact with the expression domain, resulting in binding to the RNA with an affinity of 70 ± 12 nM (Supplementary Fig. S16). We then applied our FASST method and determined a $S_{50}^{FASST}$= 54 ± 22 nM for c-di-GMP induced riboswitch conformation change. This value is two order of magnitudes higher than the binding affinity of the aptamer domain ($K_D^{ligand}$ = 980 ± 470 pM) (48). While our FASST method enabled us to observe a difference in fluorescence anisotropy and thus determine a $S_{50}^{FASST}$ value, the changes observed for this riboswitch resulted in large error bars when compared to the results obtained for SAM-I and FMN riboswitches. This discrepancy could arise from the probe design itself and could be improved by designing new probes covering the ribosomal binding site of the c-di-GMP riboswitch, varying in both length and sequence. Nevertheless, to validate our switching results, a native PAGE analysis was performed yielding $S_{50}^{NP}$ values of 24 ± 12 nM and 3 ± 5 nM, similar to the switching measured via fluorescence anisotropy. These results confirm that our FASST method can also effectively be applied to translational riboswitches. The consistent outcomes underscore the method's broad applicability for detecting structural switching across diverse riboswitch types.

## Discussion

The FASST method developed here addresses the need for a general, rapid, and high-throughput assay to screen potential drugs for their ability to induce riboswitch conformational changes. As proof of principle, we have used this method to measure switching constants for the SAM-I, FMN and c-di-GMP riboswitches binding to their native ligands and a panel of analogues.

Due to the importance of riboswitch structure and conformational changes, other approaches to measure these processes, including fluorescence-based assays, have been previously developed. One early example targeted the *glmS* ribozyme from *B. subtilis*, which was modified with fluorescein at the 5′ end, whereby ligand binding resulted in *cis* cleavage, releasing the fluorophore from the high-molecular weight RNA with a concomitant decrease in fluorescence anisotropy (72). However, this approach can only be generalized to self-cleaving ribozyme switches. In a second example, a molecular beacon approach was used to detect *B. subtilis* adenine riboswitch-mediated termination of transcription using chemically synthesized RNA probes containing fluorophores and quenchers at opposite ends (28). However, this method can only be used for riboswitches that control gene expression via transcriptional termination. Many riboswitches regulate gene expression via other mechanisms, such as modulation of translation, for instance by exposing or occluding ribosome binding sites (6), or altering mRNA stability (29), such as by exposing or occluding nuclease recognition motifs. More recently, riboswitches have been labelled with both donor and acceptor dyes via uridines to enable measurable FRET signals in single molecule studies (26,40–42). However, these strategies typically require solid-phase RNA synthesis yielding small quantities of highly expensive riboswitch constructs.

Our FASST platform offers several advantages over the above methods. First, our method makes use of stable, relatively inexpensive, ssDNA probes and unmodified, enzymatically synthesized riboswitches. While it is known that RNA–RNA hybrids form the most stable interactions, the ssDNA probes can be easily tuned for the desired binding interactions. The only design requirement is that the probes should bind with predicted melting temperatures above the temperature at which the assay will be performed (25°C in our case). Virtually all riboswitches control gene expression by either exposing or occluding RNA recognition motifs (13,73,74). In principle, fluorescent ssDNA probes can be designed to recognize these switching regions, making the FASST approach highly generalizable. Furthermore, the fluorescence anisotropy readout simplifies probe design; the sequences do not need to be self-complementary as required for molecular beacons. In addition, fluorescence anisotropy is far less sensitive to variations in probe concentration or photobleaching than fluorescence intensity or FRET-based methods, and it is highly sensitive, requiring as little as 5 nM of probe, in our hands. The FASST method also allows for low concentrations of RNA to be used, facilitating high-throughput assays. As little as 11 nM of RNA showed effective detection in our method with the HOLO^FAM-SAM^ probe (Supplementary Fig. S17). This is in stark contrast to the 1–100 μM concentrations used in the NMR, ITC (23), microarray (24), and mass spectrometry (25) approaches previously used to screen riboswitch ligands. Our methodology functions across various buffers, time points, conditions, and even in the presence of DMSO needed for low solubility ligands with limited solubility. Finally, the FASST assay is ideally suited to high throughput screening and characterization. We have demonstrated its effectiveness with 96-well plates, which can be read by a standard fluorescence plate reader in under 5 minutes. Each plate allows 48 compounds to be screened in duplicate for switching at a single concentration, or two compounds to be characterized in terms $S_{50}^{FASST}$. Thus, in principle, the method can screen over 25 000 compounds or characterize over 570 compounds in a 24-h period, using a single plate reader with the appropriate robotics to prepare samples. While this level of throughput will not always be realized, the production of RNA or the availability of compounds to test will be rate limiting rather than the FASST approach itself.

A potential limitation of our method stems from fact that we studied fully transcribed, purified, and refolded riboswitches, which makes it insensitive to transient and potentially important states that may form co-transcriptionally. A potential solution is to perform our FASST assay directly on *in vitro* transcription reactions, such that the fluorescent ssDNA probes bind directly to the RNA as it is being transcribed. This would be similar to an earlier method (28), however, RNA conformational transitions would be detected directly and ssDNA fluorescence anisotropy probes would be used rather than RNA molecular beacons, reducing the cost. Together, these important differences of our approach greatly broaden the range of RNA motifs that can be targeted and allow much lower probe concentrations to be employed. An important caveat is that the amount of RNA used in an assay should not be too far above the effective ligand affinity ${K_L}$), specifically, not more than about 5–10-fold greater than ${K_L}$. At higher riboswitch concentrations, the extracted $S_{50}^{FASST}$ values are simply equal to the half of the riboswitch concentration ${[R]_T}/2$. Under these conditions, the assay can still clearly indicate that a molecule induces switching and sets an upper bound for the true ${K_L}$ value but does not contain more precise affinity or switching information. Therefore, riboswitches that switch at low concentrations (picomolar) of ligand will be challenging to quantify due to limitations in probe concentration (5 nM) for signal detection. However, brighter fluorophores are also expected to improve the signal. Another potential limitation of our method is the vast diversity of riboswitches. While more than 55 classes of riboswitches have been described, it is estimated that thousands more remain undiscovered (13). Moreover, some of the already described classes of riboswitches contain thousands of variants (2). From all known switches, most are lacking high resolution structural information, and some do not have any obvious single-stranded regions that are unique to each conformation. In these cases, a much larger panel of probes could be designed, focusing on the expression domain and 5′ end to obtain appropriate conformational probes. In particular, the sequence of the riboswitch can be used as a template to design complementary ssDNA with ${T_m}$ values of at least 30°C. These could be screened in a SELEX (75) type manner to identify probes that bind the riboswitch in absence of ligand but not in the presence of the ligand, or vice versa.

The SAM, FMN and c-di-GMP riboswitches have been extensively studied, with numerous available structures, making them valuable for drug design. Although no drugs targeting riboswitches have been developed thus far (3,21), it has been observed that impairing cysteine biosynthesis can sensitize bacteria to antimicrobials (76). Indeed, to combat the growing problem of antimicrobial resistance (77), researchers worldwide are investigating new drugs targeting new mechanisms including riboswitches due to their wide distribution in bacteria and absence in humans and animals. Our innovative screening method holds promise for advancing the identification of potential riboswitch-targeting compounds for antimicrobial drug discovery. Specifically, our method makes use of a multi-well plate-reader format with a ratiometric fluorescent readout that can be readily implemented in a high-throughput context, to simultaneously screen hundreds of small molecules, to any riboswitch, within minutes.

In summary, we used the SAM-I, FMN and c-di-GMP riboswitches as models to develop and validate a novel screening FASST assay using ssDNA probes coupled to fluorescence anisotropy. We investigated the conformational switching of SAM-I and FMN riboswitches, revealing intriguing differences in ligand concentration requirements for triggering structural switches. Further exploration, including competitive assays with different ligands, may shed light on the underlying mechanisms and guide future drug design and screening efforts.

## Supplementary Material

gkae118_supplemental_file

## Data Availability

All data supporting the findings of this study are available in the main text or the supplementary materials. All data are available from the corresponding author upon reasonable request.
