# Supplementary material for: A sensitive and scalable fluorescence anisotropy single stranded RNA targeting approach for monitoring riboswitch conformational states
Source: Nucleic Acids Res. 2024 Feb 20;52(6):3164–79. doi: 10.1093/nar/gkae118 (PMC11014391; doi:10.1093/nar/gkae118)
Supplement: gkae118_supplemental_file [file gkae118_supplemental_file.docx]

**Supplementary information**

**A sensitive and scalable fluorescence anisotropy single stranded RNA targeting approach for monitoring riboswitch conformational states**

Maira Rivera^1^, Omma S. Ayon^1^, Suzana Diaconescu-Grabari^1^, Joshua Pottel^2^, Nicolas Moitessier^1^, Anthony Mittermaier^1*^ and Maureen McKeague^*1,3^

^1^Department of Chemistry, Faculty of Science, McGill University, Montreal, QC H3A 0B8, Canada

^2^Molecular Forecaster Inc. 910-2075 Robert Bourassa, Montreal Quebec H3A 2L1, Canada

^3^Pharmacology and Therapeutics, Faculty of Medicine and Health Sciences, McGill University, Montreal, QC H3G 1Y6, Canada

* To whom correspondence should be addressed. Email: maureen.mckeague@mcgill.ca and anthony.mittermaier@mcgill.ca

**Table of contents**

1. Tables S1-S3
2. Figures S1-S17

**Table S1. Sequences of *yitJ* SAM-I Riboswitch used**. Bold and underlined text indicate the T7 promoter region; Italic text indicate the spacer between the riboswitch and the polyA tail (bold text).

| **Name** | **DNA Sequences for T7 *in vitro* transcription 5′-3′** |
| --- | --- |
| *yitJ* SAM-I Aptamer Domain | **TTCTAATACGACTCACTATAGGG**CGTTCTTATCAAGAGAAGCAGAGGGACTGGCCCGACGAAGCTTCAGCAACCGGTGTAATGGCGATCAGCCATGACCAAGGTGCTAAATCCAGCAAGCTCGAACAGCTTGGAAGATAAGAAG*CCCCC***AAAAAAAAAAAAAAAAAAAAAAAA** |
| SAM-I Scrambled | **TTCTAATACGACTCACTATAGGG**CGTTCTTATCGTCGCGCATGCAGAAGGCATGAGGCACGAACAACGTATAGGTCTAAGCCACTACTCTCAGGTATGAGACGGCTACAAGAACTCGCGCGAAGAACAGGAACGGGATAAGAAG*CCCCC***AAAAAAAAAAAAAAAAAAAAAAAA** |
| *yitJ* SAM-I^1-156^ | **TTCTAATACGACTCACTATAGGG**ATATCCGTTCTTATCAAGAGAAGCAGAGGGACTGGCCCGACGAAGCTTCAGCAACCGGTGTAATGGCGATCAGCCATGACCAAGGTGCTAAATCCAGCAAGCTCGAACAGCTTGGAAGATAAGAAGAGACAAAATCACTGACAAAGTCTTCTTCTT*CCCCC***AAAAAAAAAAAAAAAAAAAAAAAA** |
| FMN Aptamer Domain | **TTCTAATACGACTCACTATAGGG**ATCTTCGGGGCAGGGTGAAATTCCCGACCGGTGGTATAGTCCACGAAAGTATTTGCTTTGATTTGGTGAAATTCCAAAACCGACAGTAGAGTCTGGATGAGAGAAGA*CCCCC***AAAAAAAAAAAAAAAAAAAAAAAA** |
| FMN^1-212^ | **TTCTAATACGACTCACTATAGGG**AAAAATAAATATTAAAAATAATCTTCGGGGCAGGGTGAAATTCCCGACCGGTGGTATAGTCCACGAAAGTATTTGCTTTGATTTGGTGAAATTCCAAAACCGACAGTAGAGTCTGGATGAGAGAAGAAAAGAAATTTAAGTTTTTTAACTTGTTTTCTACATTTTAGTAATCTTACCCGAATTCTATAATTCGGTTTTTTTATTTTAACTGG*CCCCC***AAAAAAAAAAAAAAAAAAAAAAAA** |
| c-di-GMP^1-209^ | **TTCTAATACGACTCACTATAGGG**GGAAAAATGTCACGCACAGGGCAAACCATTCGAAAGAGTGGGACGCAAAGCCTCCGGCCTAAACCAGAAGACATGGTAGGTAGCGGGGTTACCGATGGCAAAATGCATACACTTTGTTGACTCATCATTGACACTATGAATGCATGCTTTTGCTAATTTTCTCGGACCTGACTTGGTGGCGTATGTAACAATACTCCGAGACTAACAG*CCCCCC***AAAAAAAAAAAAAAAAAAAAAAAA** |

**Table S2.** Oligos used for PCR and Fluorescence Anisotropy experiments. Ribo means the oligo for the riboswitch; Apta for the aptamer domain, F and R are forward and reverse; and A, B, C, D refer to various oligo pieces assembled to make the full template

| **Name** | **DNA Sequences (5′-3′)** |
| --- | --- |
| **SAM-I aptamer domain PCR** | |
| SAM-I_Apta_A | TTCTAATACGACTCACTATAGGGCGTTCTTATCAAGAGAAGCAGAGGGACTGGCC |
| SAM-I_Apta_B | AGAGAAGCAGAGGGACTGGCCCGACGAAGCTTCAGCAACCGGTGTAATGGCGATCAGCCA |
| SAM-I_Apta_C | TCCAAGCTGTTCGAGCTTGCTGGATTTAGCACCTTGGTCATGGCTGATCGCCATTACAC |
| SAM-I_Apta_D | TTTTTTTTTTTTTTTTTTTTTTTTGGGGGCTTCTTATCTTCCAAGCTGTTCGAGCTTGCTGGAT |
| **SAM-I Aptamer Domain Scrambled PCR** | |
| SAM-I_SC_A | TTCTAATACGACTCACTATAGGGCGTTCTTATCGTCGCGCATGCAGAAGGCATGAGG |
| SAM-I_SC_B | GCATGCAGAAGGCATGAGGCACGAACAACGTATAGGTCTAAGCCACTACTCTCAGG |
| SAM-I_SC_C | TTCCTGTTCTTCGCGCGAGTTCTTGTAGCCGTCTCATACCTGAGAGTAGTGGCTTAGACC |
| SAM-I_SC_D | TTTTTTTTTTTTTTTTTTTTTTTTGGGGGCTTCTTATCCCGTTCCTGTTCTTCGCGCGA |
| **SAM-I^1-156^ PCR** | |
| General_A***** | TTCTAATACGACTCACTATAGGG |
| SAM-I_Ribo_B | TTCTAATACGACTCACTATAGGGATATCCGTTCTTATCAAGAGAAGCAGAGGGACTGGCCCGACGAAGCTTCAGCAACCGGTGTAATGGCGATCAGCCATGACCA |
| SAM-I_Ribo_C | TTTTTTTTTTTTTTTTTTTTTTTTGGGGGAAGAAGAAGACTTTGTCAGTGATTTTGTCTCTTCTTATCTTCCAAGCTGTTCGAGCTTGCTGGATTTAGCACCTTGGTCATGGCTGATCG |
| General_D***** | TTTTTTTTTTTTTTTTTTTTTTTTGGGG |
| **FMN aptamer domain PCR** | |
| FMN_Apta_B | TTCTAATACGACTCACTATAGGGATCTTCGGGGCAGGGTGAAATTCCCGACCGGTGGTATAGTCCACGAAAGTATTTGCTTTGATTTGGTGAAATTC |
| FMN_Apta_C | TTTTTTTTTTTTTTTTTTTTTTTTGGGGGTCTTCTCTCATCCAGACTCTACTGTCGGTTTTGGAATTTCACCAAATCAAAGC |
| **FMN^1-212^ PCR** | |
| FMN_Ribo_B | TTCTAATACGACTCACTATAGGGAAAAATAAATATTAAAAATAATCTTCGGGGCAGGGTGAAATTCCCGACCGGTGGTATAGTCCACGAAAGTATTTGCTTTGATTTGGTGAAATTCCAAAACCGACAGTAGAGTCTGGATG |
| FMN_Ribo_C | TTTTTTTTTTTTTTTTTTTTTTTTGGGGGCCAGTTAAAATAAAAAAACCGAATTATAGAATTCGGGTAAGATTACTAAAATGTAGAAAACAAGTTAAAAAACTTAAATTTCTTTTCTTCTCTCATCCAGACTCTACTGTCGGTTTTG |
| **c-di-GMP PCR** | |
| c-di-GMP_B | TTCTAATACGACTCACTATAGGGGGAAAAATGTCACGCACAGGGCAAACCATTCGAAAGAGTGGGACGCAAAGCCTCCGGCCTAAACCAGAAGACATGGTAGGTAGCGGGGTTACCGATGGCAAAATGCATACACTTTGT |
| c-di-GMP_C | TTTTTTTTTTTTTTTTTTTTTTTTGGGGGGCTGTTAGTCTCGGAGTATTGTTACATACGCCACCAAGTCAGGTCCGAGAAAATTAGCAAAAGCATGCATTCATAGTGTCAATGATGAGTCAACAAAGTGTATGCATTTTGCCATCGG |
| **ssDNA Probes** | |
| APO^FAM-SAM^ | /56-FAM/TGATAAGAACGGATAT |
| HOLO^FAM-SAM^ | /56-FAM/AAGAAGAAGACTTTGT |
| APO^FAM-FMN^ | /56-FAM/AAAAACCGAATTATAG |
| APO^FAM-c-di-GMP^ | /56-FAM/CTGTTAGTCTC |

* General A and D oligos were used for SAM-I^1-156^ and FMN^AD^ and FMN^1-212^ and c-di-GMP^1-209^ PCRs.

**Table S3. Binding affinities of aptamer domains of riboswitches used in this study.** The reported $K_{D}^{ligand}$ is the obtained from SPR measurements unless other is specified.

| RNA | Ligand | $K_{D}^{ligand}$ |
| --- | --- | --- |
| SAM-I^AD^ | SAM | 4 ± 2 nM  128 ± 27 nM^a^ |
|  | SAH | 195 ± 22 μM |
|  | Sinefungin | 129 ± 14 μM |
| FMN^AD^ | FMN | 160 ± 12 nM |
|  | Riboflavin | 30 ± 18 μM |
|  | Roseoflavin | 2 ± 1 μM |
| c-di-GMP^AD^ | c-di-GMP | 980 ± 470 pM^b^ |

^a^ $K_{D}$ measured by Isothermal titration calorimetry (ITC)

^b^ Measured using same surface plasmon resonance (SPR) assay in Chang, A.L et al. 2014 (48)

**Figure S1. Denaturing PAGE Analysis of Purified RNA Samples.** Denaturing PAGE was conducted to assess the purity of RNA samples after the final purification step. The percentage purity for each RNA is as follows: SAM-I aptamer domain (74%), SAM-I scrambled sequence (89%), SAM-I^1-156^ (100%), FMN aptamer domain (95%), FMN^1-212^ (86%), and c-di-GMP^1-209^ (93%). These purified RNA samples were subsequently employed in surface plasmon resonance (SPR), fluorescence anisotropy, and/or native PAGE experiments. Isothermal titration calorimetry (ITC) experiments exclusively utilized SAM-I riboswitch RNAs.

**Figure S2. Effect of DMSO on anisotropy change with APO^FAM-FMN^.** Fluorescence anisotropy experiment to determine the effect of several DMSO concentrations on anisotropy using APO^FAM-FMN^ probe. For each percentage of DMSO, the assay includes 5 nM APO^FAM-FMN^ probe and 350 nM of FMN^1-212^ riboswitch with (60 μM) and without FMN. It showed higher anisotropy without FMN indicating the APO^FAM-FMN^ probe binds to the unbound state of the riboswitch (black) and there has been no significant change in the anisotropy caused by increasing % of DMSO. Error bars shown here are for technical triplicates.

**Figure S3. ITC of SAM-I^AD^ and SAM-I^1-156^**. Experiment performed with 10 μM of SAM-I^AD^, 8.8 μM SAM-I^1-156^ with 105 μM SAM. First injection of 0.2 μL by 0.4 s with 60 s spacing; injections 2-19 of 2 μL by 4 s and with 180 s of spacing; 20^th^ injection of 1.6 μL by 4 s with 180 s spacing, reference power of 7.00 μcal/s. One set of sites binding model was used for the fit to obtain a $K_{D}$ of 128 ± 27 nM for SAM-I^AD^, with a $\Delta H$ of -25.3 ± 0.7 kcal/mol and a $N$ value of 0.537 ± 0.009. Considering that the purity of SAM-I^AD^ RNA is close to 60%, the $N$ value can be corrected to 0.94 ± 0.02.


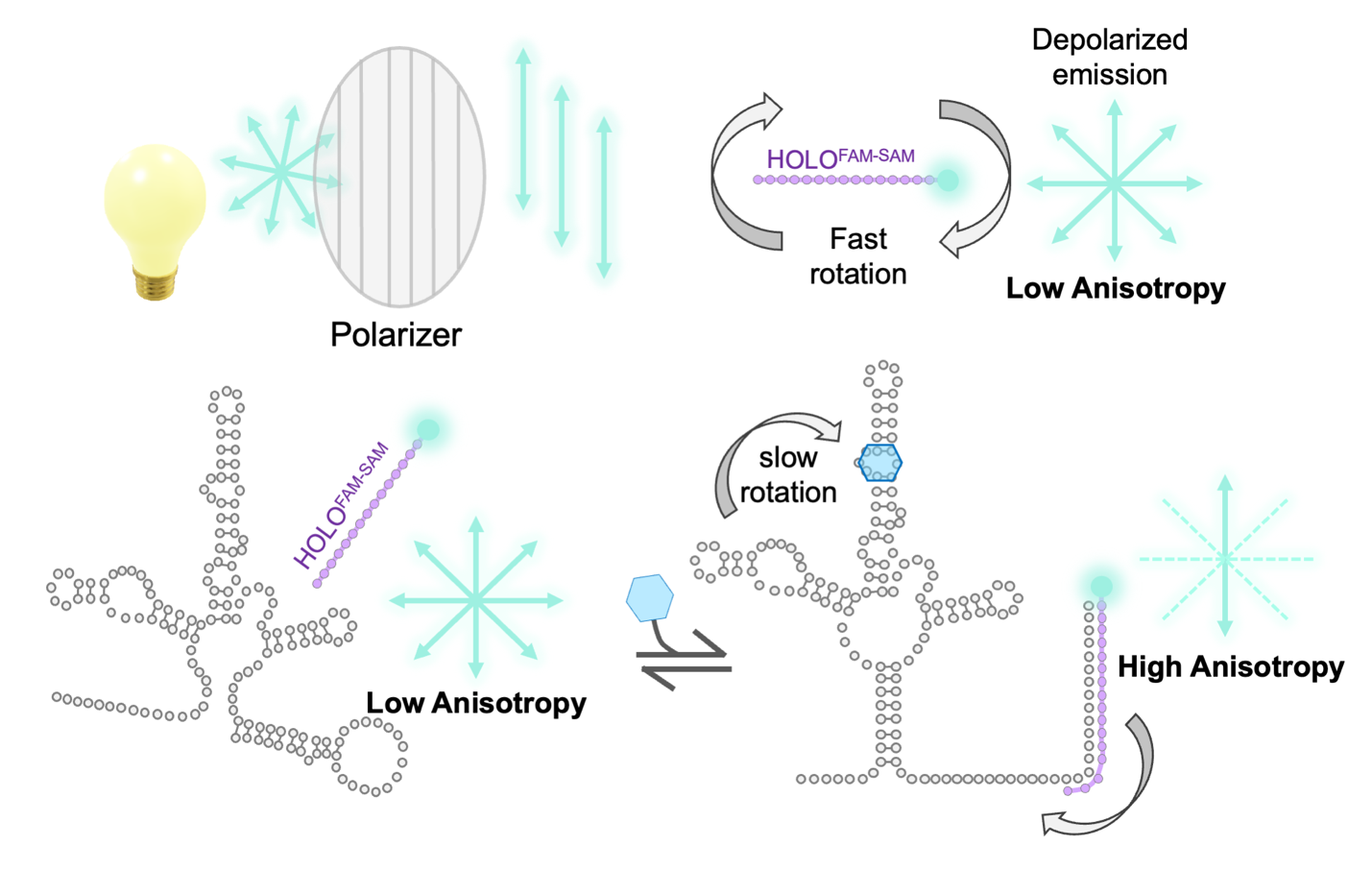


**Figure S4. Fluorescence anisotropy approach with the HOLO^FAM-SAM^ probe.** The design of the ssDNA probes aimed to target single stranded RNA regions of the riboswitches exposed in either the apo or holo state. Here as an example with the HOLO probe, when polarized light is applied to the fluorescently labeled probe, it exhibits depolarized emission, and therefore a low anisotropy. This is attributed to the fast tumbling in solution. However, when the binding site for the HOLO probe is available in the riboswitch due to the structural switch triggered by SAM, fluorescence emission from the probe becomes more polarized. This polarization arises from the slower rotation of the complex formed between the fluorescent probe and the RNA, resulting in high anisotropy values.

**Figure S5. Binding of the fluorescently labelled ssDNA probe to the Riboswitch**. Determining the binding of the probes using fluorescence anisotropy upon increasing concentrations of the riboswitch. Increasing anisotropy indicates the probe binds to the riboswitch. **(A)** APO^FAM-SAM^ binds to both SAM bound (black) and unbound (teal) states, however it prefers to bind to the apo state with 2-fold higher affinity. **(B)** HOLO^FAM-SAM^ binds to the riboswitch in presence of SAM (black) whereas it showed close to zero anisotropy in the absence of SAM (purple). **(C)** APO^FAM-FMN^ probe binds to the riboswitch (teal), in presence of FMN it showed nearly zero anisotropy indicating no binding to the FMN bound state (black). Error bars shown represent the standard deviation for technical duplicates and solid lines correspond to a one-site binding fit.

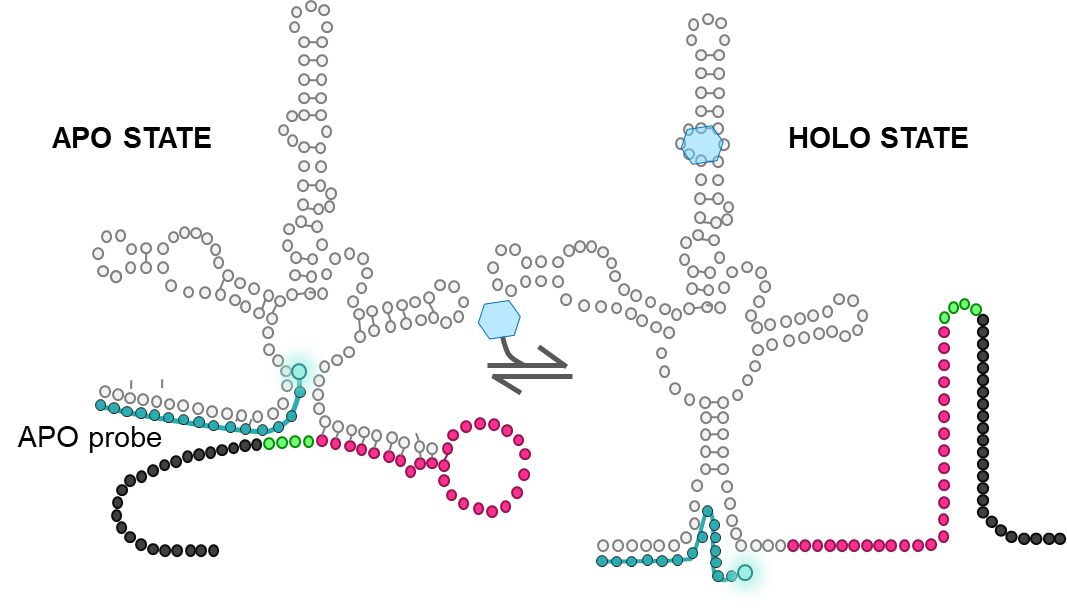


**Figure S6. Lack of binding of APO^FAM-SAM^ to SAM-I^AD^ and possible binding of the probe to the HOLO state of SAM-I^1-156^.** Experiments performed under the same experimental conditions as for SAM-I^1-156^ were performed with SAM-I^AD^ which revealed no changes in anisotropy with increasing concentrations of SAM, suggesting that the probe is not able to bind to other regions in the riboswitch. Therefore, a model of potential binding to the HOLO state is illustrated (bottom).


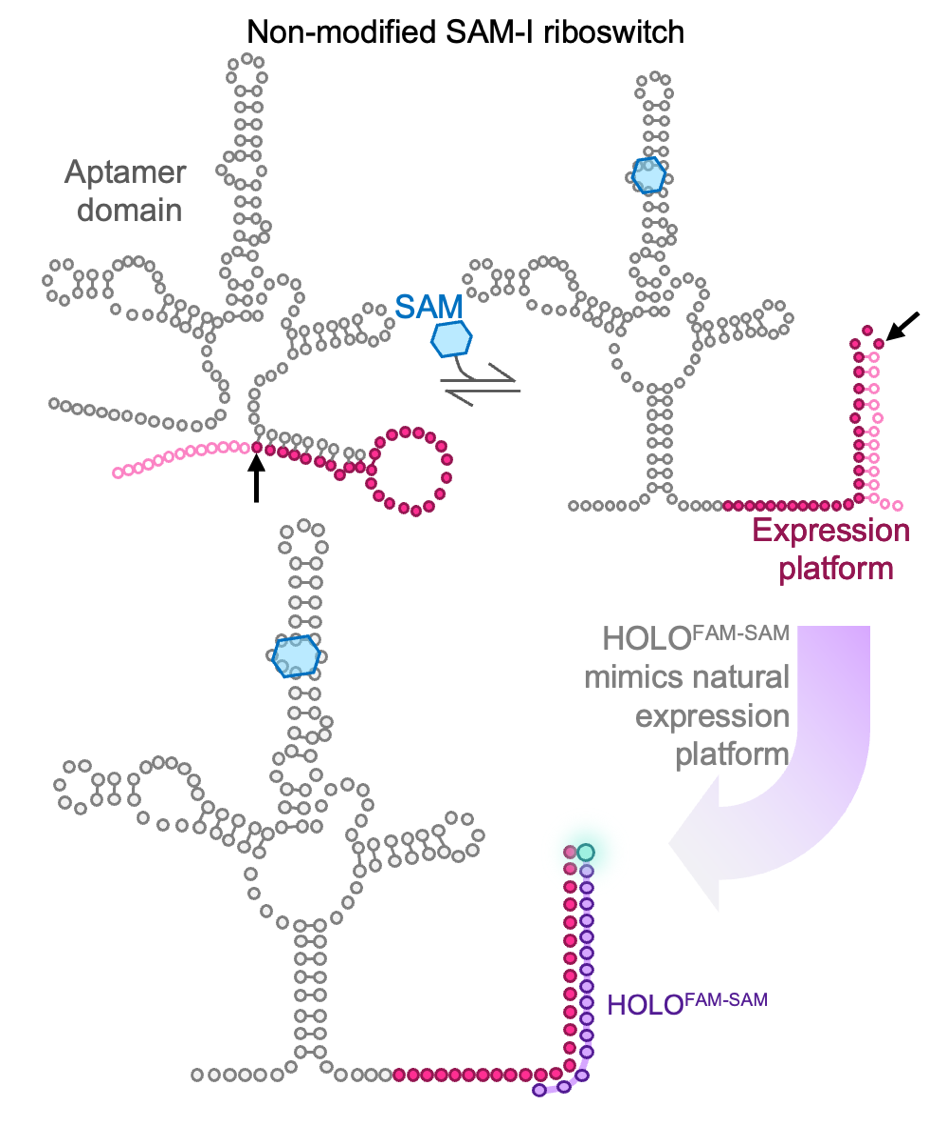


**Figure S7. The HOLO^FAM-SAM^ probe mimics the natural expression platform of the SAM-I riboswitch.** Black arrow indicates where our SAM-I riboswitch constructure was truncated, resulting in an impaired expression platform.

**Figure S8. Impact of the poly(A) tail on SAM-I^1-156^ switching**. Fluorescence anisotropy experiments were performed in the same experimental conditions as our SAM-I riboswitch construct (Table 1) using a version of the RNA that lacks the spacer and poly(A) tail (see **Fig. 2**). Similar $S_{50}^{FASST}$ values were obtained (**Table 2**) suggesting that the presence of the poly(A) tail does not impact the binding and switching of the SAM-I riboswitch.


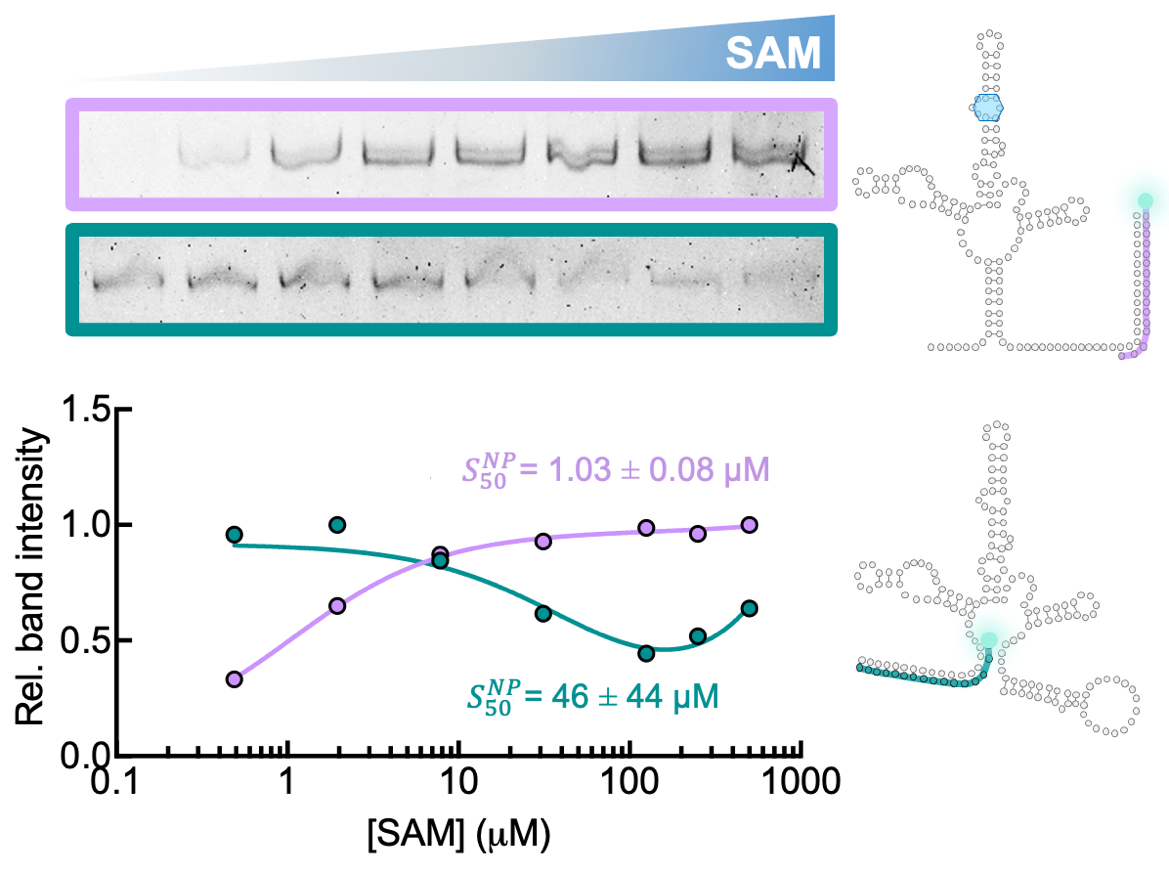


**Figure S9. HOLO^FAM-SAM^ and APO^FAM-SAM^ probes binding to SAM-I riboswitch followed by native PAGE**. From the fluorescence anisotropy experiments, samples were loaded onto a 10% native PAGE. The band intensity was analyzed with the Image Lab software (BioRad) and their relative value was determined with respect to the highest value. Switching constants were determined by fitting a one site binding model using GraphPad Prism; the error was determined from the fit.

**
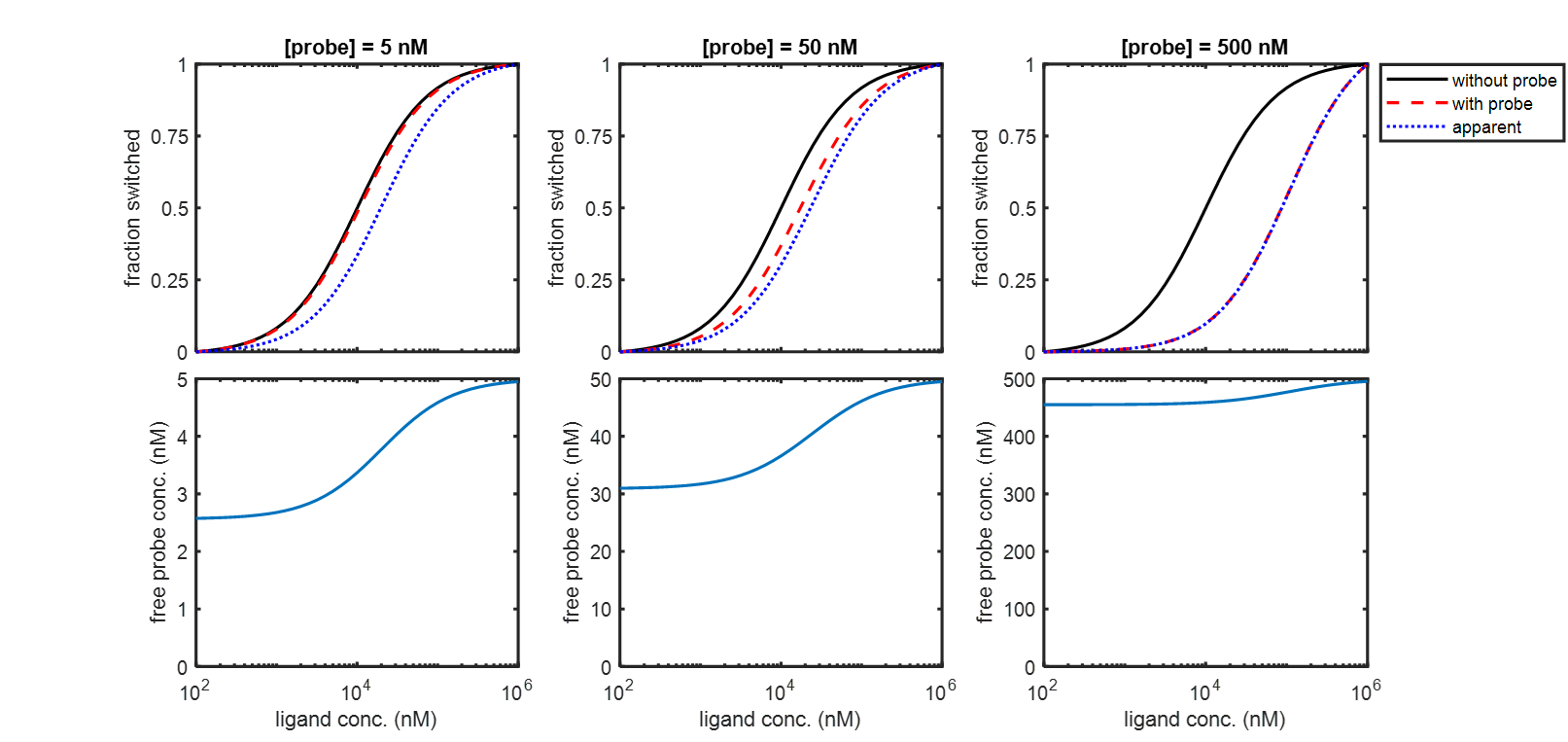
**

**Figure S10.** Calculated ligand binding curves for a riboswitch with an APO^FAM^ probe. The top panels show the fraction of riboswitch molecules in the holo state ($\frac{\left[ HL \right]+\left[ HLP \right]}{\left[ R \right]_{T}}$) calculated with $\left[ P \right]_{T}$=0 (black solid lines) and $\left[ P \right]_{T}$≠0 (red dashed lines). Also shown is the apparent switching curve as would be detected by the fluorescent probe ($\frac{\left[ P \right]-\left[ P \right]_{init}}{\left[ P \right]_{final}-\left[ P \right]_{init}}$), where $\left[ P \right]_{init}$ and $\left[ P \right]_{final}$ are the concentrations of free probe at the beginning and end of the titration (blue dotted line). The bottom panels show the concentrations of free probe as a function of ligand concentration. Calculations were performed with $\left[ R \right]_{T}$=50 nM, $K_{L}$=10 μM, $K_{P}^{apo}$=50 nM, and no binding of probe to the holo form. $\left[ P \right]_{T}$ was equal to 5, 50, and 500 nM in the left, middle, and right panels.


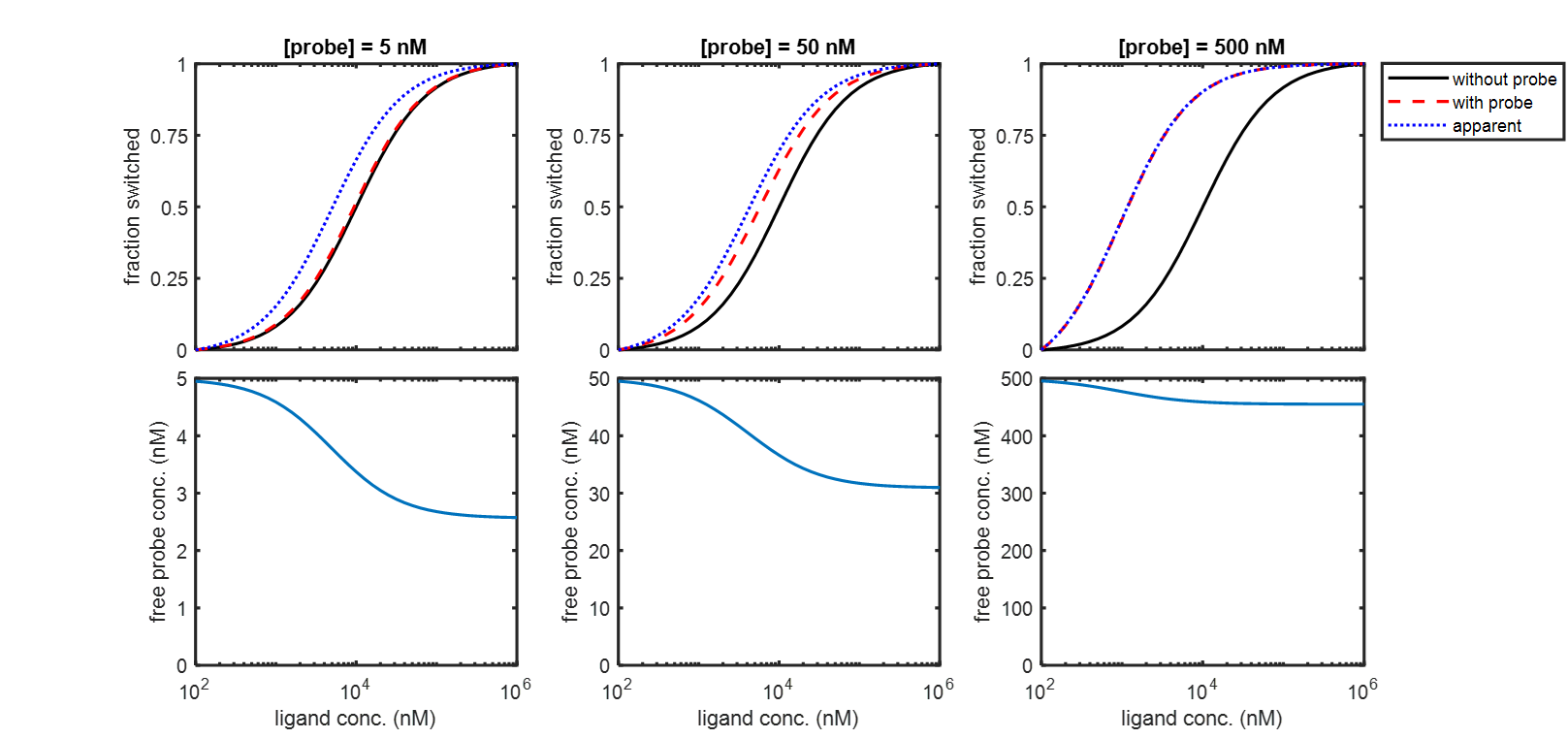


**Figure S11.** Calculated ligand binding curves for a riboswitch with a HOLO^FAM^ probe. The top panels show the fraction of riboswitch molecules in the holo state ($\frac{\left[ HL \right]+\left[ HLP \right]}{\left[ R \right]_{T}}$) calculated with $\left[ P \right]_{T}$=0 (black solid lines) and $\left[ P \right]_{T}$≠0 (red dashed lines). Also shown is the apparent switching curve as would be detected by the fluorescent probe ($\frac{\left[ P \right]-\left[ P \right]_{init}}{\left[ P \right]_{final}-\left[ P \right]_{init}}$), where $\left[ P \right]_{init}$ and $\left[ P \right]_{final}$ are the concentrations of free probe at the beginning and end of the titration (blue dotted line). The bottom panels show the concentrations of free probe as a function of ligand concentration. Calculations were performed with $\left[ R \right]_{T}$=50 nM, $K_{L}$=10 μM, $K_{P}^{holo}$=50 nM, and no binding of probe to the apo form. $\left[ P \right]_{T}$ was equal to 5, 50, and 500 nM in the left, middle, and right panels.


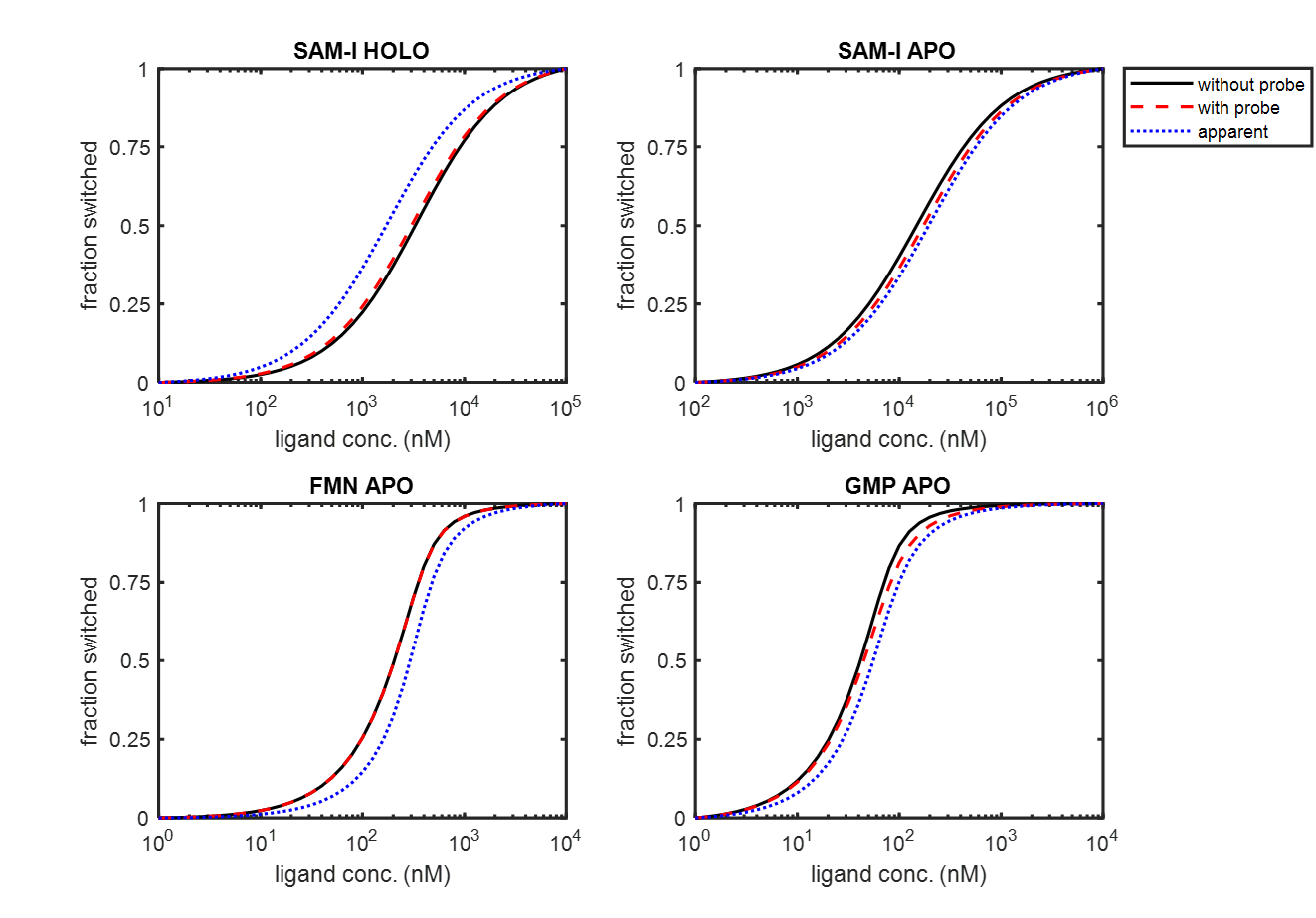


**Figure S12.** Calculated ligand binding curves for riboswitches used in this study. Black solid, red dashed, and blue dotted lines have the same meaning as in Figures S8 and S9. For **SAM-I** **HOLO** $\left[ R \right]_{T}$=350 nM, $\left[ P \right]_{T}$=50 nM, $K_{L}$=3.3 μM, $K_{P}^{holo}$=350 nM, and no probe binding to the apo state), the true $S_{50}$ value (ligand concentration for 50% switching, black solid line) was 3.3 μM and the apparent $S_{50}$ value (blue dash-dot line) was 1.7 μM. For **SAM-I APO** ($\left[ R \right]_{T}$=75 nM, $\left[ P \right]_{T}$=50 nM, $K_{L}$=15 μM, $K_{P}^{apo}$=75 nM, $K_{P}^{holo}$=150 nM), the true and apparent $S_{50}$ value were 15 and 19 μM. For **FMN APO** (($\left[ R \right]_{T}$=350 nm., $\left[ P \right]_{T}$=5 nM, $K_{L}$=30 nM, $K_{P}^{apo}$=340 nM, and no probe binding to the holo state) the true and apparent $S_{50}$ values were 200 and 290 nM. For **GMP APO** (($\left[ R \right]_{T}$=70 nM, $\left[ P \right]_{T}$=50 nM, $K_{L}$=6 nM, $K_{P}^{apo}$=70 nM, and no probe binding to the holo state) the true and apparent $S_{50}$ values were 42 and 56 nM.


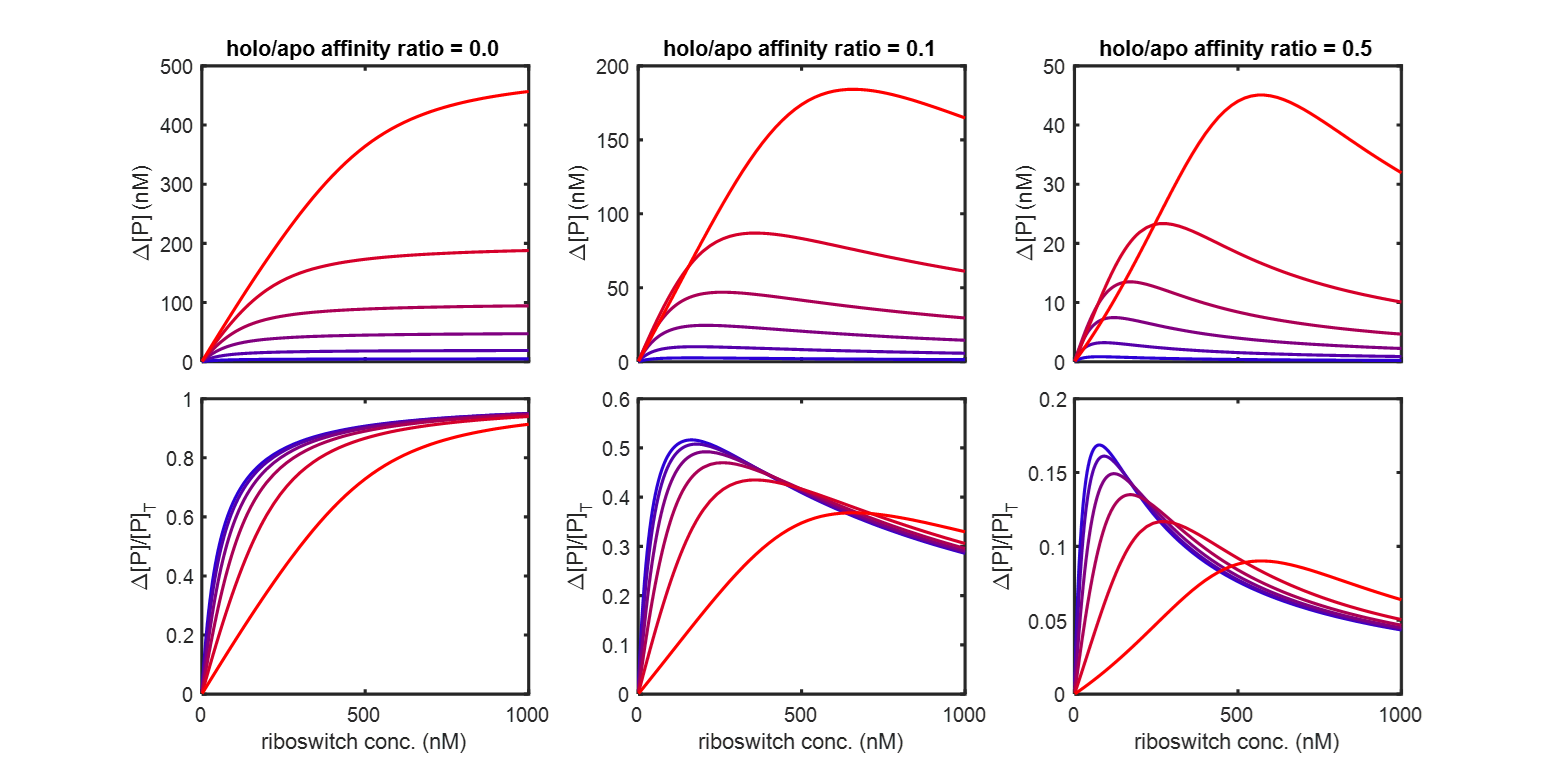


**Figure S13.** Calculated differences in free probe concentration between the absence of ligand ($\left[ L \right]_{T}$=0) and the presence of saturating ligand ($\left[ L \right]_{T}$= 100 mM) for probes that bind both the apo and holo forms. The differences in probe concentrations are directly proportional to the sensitivity of the experiment. The top panels indicate the overall changes in free probe concentration ($\left[ P \right]_{final}-\left[ P \right]_{init}$) and the bottom panels show relative changes in concentration ($\frac{\left[ P \right]_{final}-\left[ P \right]_{init}}{\left[ P \right]_{T}}$). Curves were calculated with $K_{L}$=10 μM, $K_{P}^{apo}$=50 nM, $\left[ P \right]_{T}$= 5 (blue), 20, 50, 100, 200, 500 (red) nM. The left panels were calculated with no holo state binding, the middle and left panels were calculated with $K_{P}^{holo}$=500 and 100 nM, respectively.


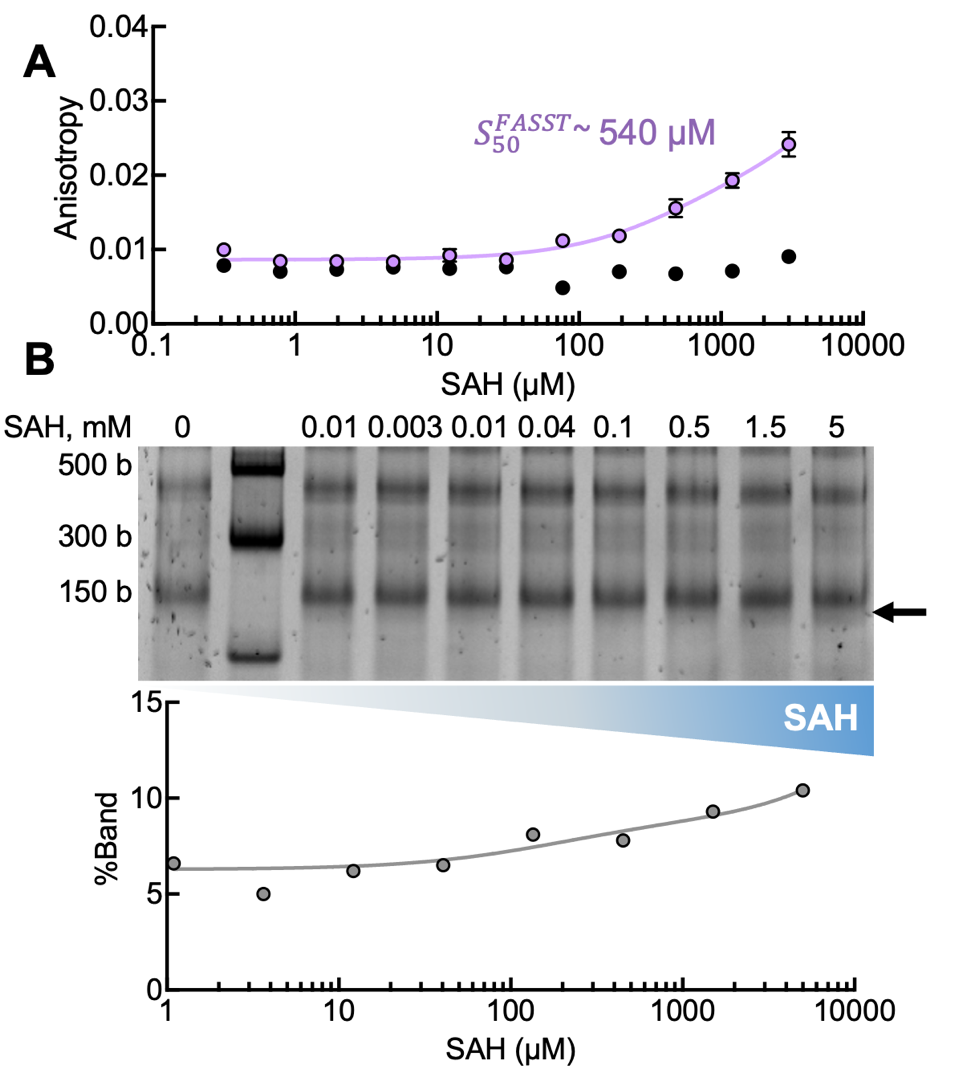


**Figure S14. SAM-I switching triggered by SAH with 5 mM MgCl_2_. (A)** After overnight incubation with SAH in the presence of 5 mM MgCl_2_, switching was detected with the HOLO^FAM-SAM^ probe with a $S_{50}^{FASST}$ around 540 µM. **(B)** In the absence of probes, gel analysis indicates a different band pattern when the riboswitch is incubated with SAH concentrations ≥ 0.5 mM. This observation was confirmed by determining the intensity of the band indicated by the black arrow and is in good agreement with the switching detected with the ssDNA probe. Error bars correspond to the standard deviation from technical duplicates.

**Fig. S15. Analogue affinities to the aptamer domains of SAM-I and FMN riboswitches.** SPR sensorgrams (left) and affinity analysis at equilibrium (right) performed with technical triplicates and duplicates for SAM-I and FMN riboswitches, respectively. **(A** and **B)** SAM analogues were tested with SAM-I^AD^ resulting in 195 ± 22 µM and 129 ± 14 µM for SAH and SF, respectively. **(C** and **D)** FMN analogues binding to FMN^AD^ was assessed resulting in 30 ± 18 µM and 2± 1 µM for RF and RoF, respectively. Error bars correspond to the standard deviation from technical triplicates.


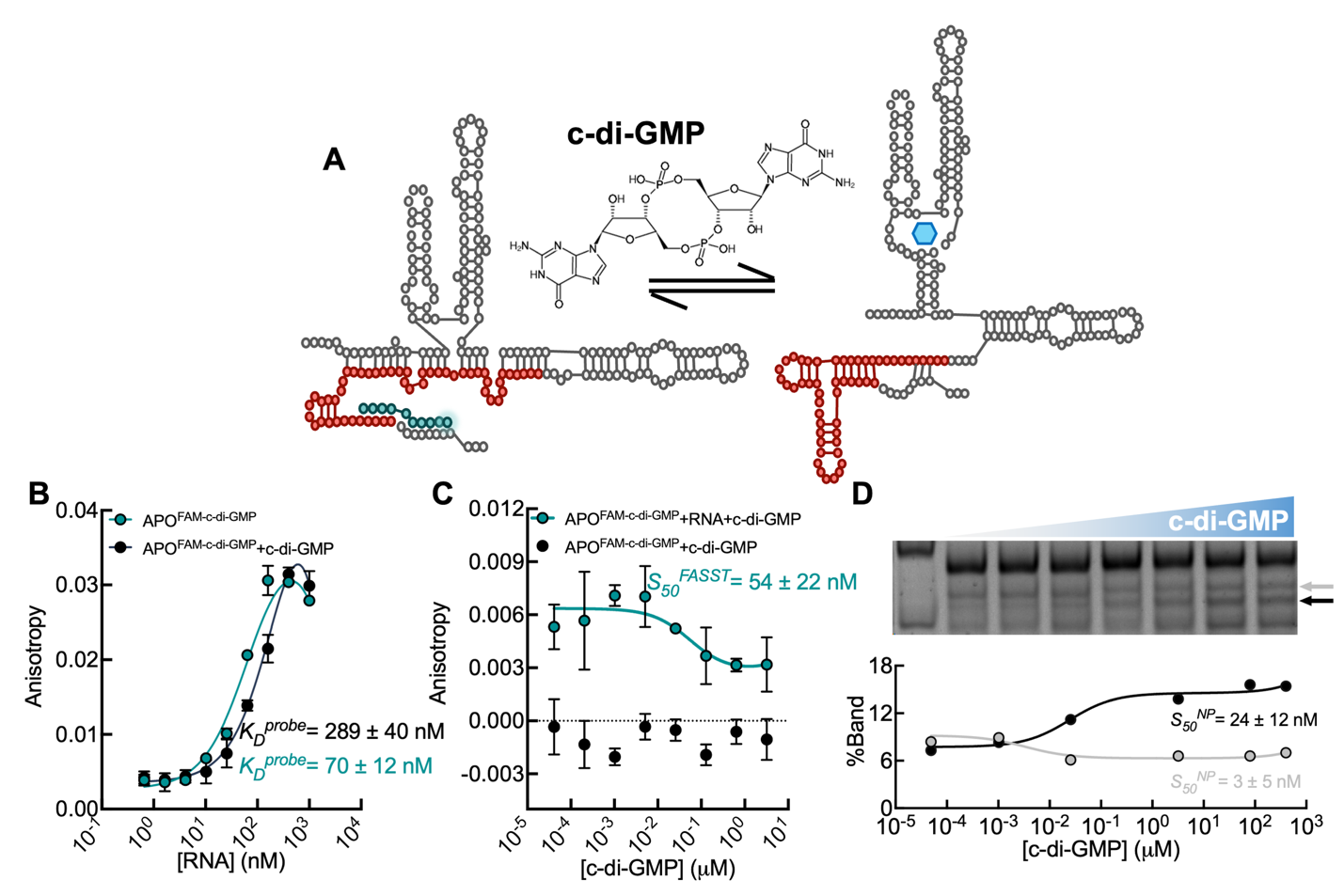


**Figure S16. c-di-GMP riboswitch switching evaluated by fluorescence anisotropy and native PAGE**. (A) Secondary structure representation of the c-di-GMP riboswitch and the binding site of the probe in the apo state, in red is the expression platform. (B) RNA calibration curve using the APO^FAM-c-di-GMP^ probe at a constant concentration of 50 nM. (C) Fluorescence anisotropy-based switching experiment conducted at an RNA concentration of 70 nM, revealing switching constants $S_{50}^{FASST}$in the nanomolar range. (D) Native PAGE switching assay band quantification, indicating switching constants
$\boldsymbol{S}_{\boldsymbol{50}}^{\boldsymbol{NP}}$in the nanomolar range.

**Figure S17. SAM-I with 5 nM ssDNA probes. (A)** RNA calibration curve of SAM-I riboswitch RNA binding to HOLO^FAM-SAM^ and APO^FAM-SAM^ probes at 5 nM. **(B)** SAM-triggered switching using 22 nM riboswitch and 5 nM of the HOLO^FAM-SAM^ probe **(C)** SAM-triggered switching using 11 nM riboswitch and 5 nM of the APO^FAM-SAM^ probe. Error bars corresponded to standard deviation from technical duplicates. In B and C, black dots correspond to the probe in the presence of increasing SAM concentrations.
